# The Expression Patterns and Prognostic Value of the Proteasome Activator Subunit Gene Family in Gastric Cancer Based on Integrated Analysis

**DOI:** 10.3389/fcell.2021.663001

**Published:** 2021-09-28

**Authors:** Yongdong Guo, Xiaoping Dong, Jing Jin, Yutong He

**Affiliations:** Cancer Institute, Fourth Hospital of Hebei Medical University, Shijiazhuang, China

**Keywords:** proteasome activator subunit, Kaplan–Meier plotter, gastric cancer, prognosis, biomarkers, immune infiltration

## Abstract

Increasing evidence supports that proteasome activator subunit (PSME) genes play an indispensable role in multiple tumors. The diverse expression patterns, prognostic value, underlying mechanism, and the role in the immunotherapy of *PSME* genes in gastric cancer (GC) have yet to be fully elucidated. We systematically demonstrated the functions of these genes in GC using various large databases, unbiased *in silico* approaches, and experimental validation. We found that the median expression levels of all *PSME* genes were significantly higher in GC tissues than in normal tissues. Our findings showed that up-regulated *PSME1* and *PSME2* expression significantly correlated with favorable overall survival, post-progression survival, and first progression survival in GC patients. The expression of *PSME1* and *PSME2* was positively correlated with the infiltration of most immune cells and the activation of anti-cancer immunity cycle steps. Moreover, GC patients with high *PSME1* and *PSME2* expression have higher immunophenoscore and tumor mutational burden. In addition, a receiver operating characteristic analysis suggested that *PSME3* and *PSME4* had high diagnostic performance for distinguishing GC patients from healthy individuals. Moreover, our further analysis indicated that *PSME* genes exert an essential role in GC, and the present study indicated that *PSME1* and *PSME2* may be potential prognostic markers for enhancing survival and prognostic accuracy in GC patients and may even act as potential biomarkers for GC patients indicating a response to immunotherapy. *PSME3* may serve as an oncogene in tumorigenesis and may be a promising therapeutic target for GC. PSME4 had excellent diagnostic performance and could serve as a good diagnostic indicator for GC.

## Introduction

Gastric cancer (GC) is one of the most prevalent solid tumors and the second most frequent cause of cancer-related deaths worldwide (Bray et al., 2018; Cavatorta et al., 2018). Although the treatment for GC has advanced greatly in recent years, the prognosis is still unfavorable for most patients, mainly because they are still initially diagnosed at an advanced stage with lymphatic or distant metastasis (Nishida and Doi, 2014; Park et al., 2018; Miller et al., 2019). The recent development of immune checkpoint blockpoint blockade (ICB) therapy has revolutionized the field of cancer therapy. ICB permits that the patient’s immune system recognizes and attacks cancer cells, mounting an effective antitumoral response that contributes to eradicate the disease. Nevertheless, only up to two thirds of cancer patients benefit from immunotherapy, highlighting the need of discovering new biomarkers to select patients suitable for ICB (Jiang et al., 2018). Given the limited therapeutic approaches and the lack of prognostic and therapeutic biomarkers for advanced GC, it is imperative to search for novel biomarkers that can inform about the immunotherapy response of the patient, guiding clinicians to choose the most suitable therapy strategy, and to help researchers to develop new therapeutic targets for GC.

Proteasomes are important compartmentalized proteases present in all eukaryotes and archaea (Rechsteiner and Hill, 2005). Protein degradation mediated by the proteasome is essential for protein homeostasis and is critically dependent on proteasome activator subunit (*PSME*) genes, mainly consisting of *PSME1*, *PSME2*, *PSME3*, and *PSME4* (which encode PA28α, PA28β, PA28γ/REGγ, and PA200, respectively; Peters et al., 1994; Masters et al., 2005). Multiple studies have showed that proteasome activators not only balance proteasome function but also correlate with multiple malignancies and act as prognostic predictors (Sanchez-Martin et al., 2013; Wang et al., 2019; Yu et al., 2019). Additionally, several studies have reported that *PSME1* is dysregulated in several different cancers, including prostate cancer and oral squamous cell carcinoma (OSCC), suggesting that *PSME1* may act as a novel prognostic factor (Lemaire et al., 2007; Longuespee et al., 2012; Feng et al., 2016). PSME2 is significantly down-regulated in esophageal carcinoma tissues compared to normal tissues and could act as a potential tumor inhibitor (Chen J. Y. et al., 2017). PSME3 is significantly up-regulated in breast cancer (Chai et al., 2014, 2015) and in OSCC (Li J. et al., 2015) and plays an essential role in tumorigenesis. The overexpression of *PSME3* has been significantly associated with unfavorable overall survival (OS). Finally, *PSME4* modulates radiation sensitivity impacting glutamine metabolism to improve the survival of cervical carcinoma cells after radiation exposure (Blickwedehl et al., 2012). Despite these indications about the dysregulated expression patterns and clinical significance of *PSME* family genes, as well as their potential involvement in GC, the role of *PSME* family genes in cancer has not yet been systematically demonstrated.

In this study, we analyzed the correlation between the expression levels of *PSME* family genes and different clinicopathological features, including clinical stage, historical subtypes, nodal metastasis status, and *Helicobacter pylori* infection status, as well as prognostic values and genetic and epigenetic alterations. Additionally, we explored the role of these genes in the prediction of immunotherapeutic benefits and the expression levels of these genes in GC at the single-cell level. For these analysis, we used multiple large databases, including STRING; Database for Annotation, Visualization, and Integrated Discovery (DAVID); RMBase; cBioPortal; Tumor Immune Single Cell Hub (TISCH); muTarget; GSCALite; The Cancer Genome Atlas (TCGA); Gene Expression Omnibus (GEO); LinkedOmics; UALCAN; and Kaplan–Meier plotter, and bioinformatics approaches. We hope that our findings may provide key genes to improve therapeutic outcomes and enhance the accuracy of prognosis for patients with GC.

## Materials and Methods

### Patients and Samples

Forty patients from the Fourth Hospital of Hebei Medical University were included in the present study (Supplementary Table 1). All patients were surgically treated at the Fourth Hospital of Hebei Medical University from January 1, 2018, to December 31, 2019. The inclusion criterion was that the patients received a pathological diagnosis of primary GC. Written informed consent was obtained from all patients, and the study was approved by the Ethics Committee of the Fourth Affiliated Hospital of Hebei Medical University.

### RNA Isolation and Reverse Transcription-Quantitative Polymerase Chain Reaction

Total RNA was extracted from frozen tumor samples and corresponding non-tumorous tissues using TRIzol reagent (Invitrogen, Thermo Fisher Scientific, Inc.). Power SYBR Green PCR Master Mix (Ribobio, Guangzhou, China) was used as the amplification reaction mixture, following manufacturer’s instructions. The primers and reaction conditions for the *PSME* genes are listed in Supplementary Table 2. Human *GAPDH* gene was used as an internal control. The relative expression levels of *PSME* genes were calculated using the 2^–ΔΔ*CT*^ method, as the previously described (Livak and Schmittgen, 2001).

### Data Source

The Cancer Genome Atlas is a large repository that contains high-throughput data of more than 30 human cohort cancer studies (Tomczak et al., 2015). The expression profiles of the *PSME* genes were obtained from the XENA database^1^. Detailed information on the GEO datasets used in this study is provided in Supplementary Table 3. Tumor Immune Estimation Resource (TIMER)^2^ is an open-source database that permits to explore and visualize the correlation between immune infiltrates and gene expression, clinical outcome, and other prognosis-related parameters, with over 10,897 tumor samples from 32 tumor types (Li T. et al., 2017; Li T. et al., 2020). The Diff-Exp module of the TIMER database was used to evaluate the expression of *PSME* genes between tumor and normal tissues across all tumor types in TCGA database. The expression levels of *PSME* genes in the different clinicopathological statuses of patients with GC were assessed using UALCAN database (Chandrashekar et al., 2017).

### Kaplan–Meier Plotter Analysis

In order to evaluate the diagnostic value of *PSME* genes in GC patients, a receiver operating characteristic (ROC) curve was constructed using the pROC package in R software (Robin et al., 2011). We evaluated OS, post-progression survival (PPS), and first progression survival (FPS) using the Kaplan−Meier plotter database^3^ based on the best-performing threshold of these genes (Szász et al., 2016). Moreover, we also evaluated the correlation between expression levels of *PSME* genes and prognosis of GC patients with different clinicopathological characteristics, such as gender, Lauren classification, degree of differentiation, human epidermal growth factor receptor 2 (HER2) status, and different types of treatment.

### Alterations and Epigenetic Modifications Analysis

The cBioPortal for Cancer Genomics^4^ is a user-friendly platform that provides large-scale tumor genomics data sets for exploration and analysis (Cerami et al., 2012; Gao et al., 2013). The frequency of *PSME* gene alterations in patients with GC was evaluated using this resource. muTarget^5^ is a cancer biomarker/target discovery tool that can be used for studying new drug targets in a cohort of patients with a given mutations (Nagy and Gyorffy, 2020). In the present study, we used muTarget to identify *PSME* gene expression changes related to gene mutations and to identify mutations altering the expression of *PSME* genes. RMBase is a user-friendly database that integrates epitranscriptome sequencing data to evaluate post-transcriptional modifications of genes (Sun W. J. et al., 2016).

### Functional Enrichment and Correlation Analysis

Relationships among individual expression of *PSME* genes were evaluated using the Pearson correlation coefficient and performed using the corrplot (Taiyun Wei and Simko, 2017) package in R software. Gene Ontology (GO) and Kyoto Encyclopedia of Genes and Genomes (KEGG) enrichment analyses were conducted using the DAVID (Huang da et al., 2009)^6^. Gene Set Enrichment Analysis (GSEA) of *PSME* family genes were annotated using *PSME*-correlated genes using the Pearson correlation test in STAD cohorts obtained from TCGA database and gene sets (h.all.v6.2.entrez.gmt) obtained from the Molecular Signatures Database using GSEA V3.0^7^ (Subramanian et al., 2005) and clusterProfiler (Yu et al., 2012) package in R software. We also used the LinkedOmics database (Vasaikar et al., 2018) to evaluate the differentially expressed genes related to *PSME* genes in the GC cohort. Then, overrepresentation enrichment analysis was utilized to analyze the presence of members of the reactome pathway and kinase target in the set of genes defined as *PSME*-associated. Enrichment results with *P* < 0.05 and false discovery rate < 0.05 were visualized using the clusterProfiler package.

### GSCALite Database

GSCALite^8^ is a comprehensive omics data analysis platform for gene-set cancer analysis (Liu et al., 2018). Abnormal expression of genes affects clinical responses to therapy and can be used for drug screening. In our study, GSCALite was used to analyze the copy number variation profile of *PSME* genes in STAD. Drug sensitivity and the expression of *PSME* genes were explored by Spearman correlation analysis with IC50 based on the cancer therapeutic response portal.

### Immune Infiltration Analysis

The infiltration levels of immune cell types were assessed by the single-sample gene set enrichment analysis (ssGSEA) method in R software (Hänzelmann et al., 2013). ssGSEA applies gene signatures expressed by immune cell populations to indivaduals (Barbie et al., 2009). In this study, 24 immune cell types were analyzed using the deconvolution approach (Bindea et al., 2013). Furthermore, we explored the correlation between *PSME* and immune cell infiltration. Immune-related genes were obtained from a previously published paper (Li G. et al., 2017). The immunoscore for every STAD patient was calculated using ESTIMATE algorithm using the “estimate” package in R software (Yoshihara et al., 2013). We also calculated immunophenoscore (IPS) to infer the potential response of STAD patients to immunotherapy (Charoentong et al., 2017).

### Tumor Immune Single Cell Hub Satabase

Tumor Immune Single Cell Hub^9^ is an interactive and online database that integrates single-cell transcriptomic profiles of million cells from multiple high-quality cancer datasets across nearly all cancer types (Sun et al., 2020).

### Statistical Analysis

Statistical analysis was performed using R (version, 4.0.4) and SPSS 21.0. The results of real-time reverse transcription-quantitative polymerase chain reaction (RT-qPCR) are shown as the mean ± S.D. Student’s test and Wilcoxon test were used to compare the expression between different groups. *P* < 0.05 was considered as statistically significant.

## Results

### Relative Expression Levels of *PSME* Family Members in Patients With Gastric Cancer

**FIGURE 1:**
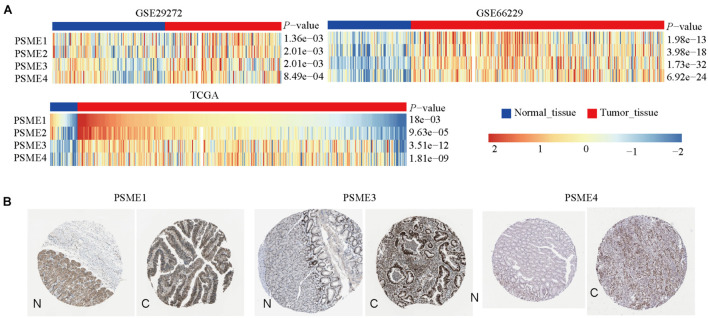
The transcriptional and protein expression of proteasome activator subunit (PSME) family genes in patients with gastric cancer (GC). **(A)** High transcriptional expression of PSME family genes in GC tissues compared to normal tissues [Gene Expression Omnibus (GEO) and The Cancer Genome Atlas (TCGA)]. **(B)** High protein expression of PSME1, PSME3, and PSME4 were obtained in tumor tissues (The Human Protein Atlas). C, cancer; N, normal tissues.

**FIGURE 2:**
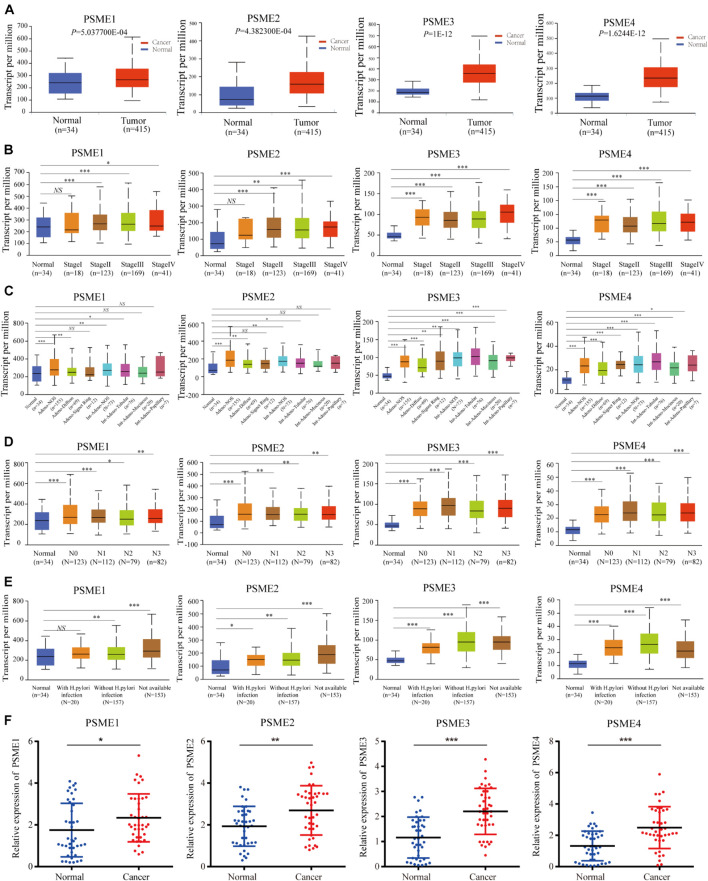
Transcriptional expression level of PSME family genes in GC patients with different clinicopathological features. **(A)** Transcriptional expression level of PSME family genes between the GC tissues and non-cancerous tissues using UALCAN database. **(B)** Differences of PSME genes’ expression among tumor stages. **(C)** Differences of PSME genes’ expression among histological subtypes (AdenoNOS, adenocarcinoma NOS; AdenoDiffuse, adenocarcinoma diffuse; Adeno SignetRing, adenocarcinoma signet ring; IntAdenoNOS, intestinal adenocarcinoma NOS; IntAdenoTubular, intestinal adenocarcinoma tubular; IntAdenoMucinous, intestinal adenocarcinoma mucinous; and IntAdenoPpillary, intestinal adenocarcinoma papillary). **(D)** Differences of PSME genes’ expression among nodal metastasis status. N0, no regional lymph node metastasis; N1, metastases in 1 to 3 axillary lymph nodes; N2, metastases in 4 to 9 axillary lymph nodes; N3, metastases in 10 or more axillary lymph nodes. **(E)** Differences of PSME genes’ expression among *H. pylori* infection status. **(F)** Validated expression of PSME family genes in 40 patients with GC. *P < 0.05, **P < 0.01, and ***P < 0.001; *NS* indicates no statistical significance.

We compared the mRNA expression level of *PSME* genes between tumor and normal tissues across all cancer types in TCGA by using the TIMER database. The result showed that *PSME* family genes were dysregulated in most cancers, including breast cancer, colon cancer, esophageal cancer, lung carcinoma, hepatic carcinoma, and GC (Supplementary Figure 1). We further explored the mRNA and protein expression level of *PSME* family genes in GC tissues based on GEO, TCGA, and The Human Protein Atlas (HPA) databases. The results uncovered that *PSME* genes were highly expressed in GC tissues compared to non-cancerous tissues (Figure 1). Next, we evaluated the transcriptional expression of *PSME* genes in gastric tumor tissues and normal tissues by using the UALCAN database. The results indicated that transcriptional expression of all *PSME* genes is overexpressed in tumor tissues compared to non-cancerous tissues in patients with GC (Figure 2A), consistent with the validated expression of *PSME* family genes in 40 cases with GC using RT-qPCR (Figure 2F). Additionally, we assessed the expression level of PSME family genes in 37 GC cell lines (Supplementary Figure 2). The expression level of all *PSME* genes was significantly correlated with tumor stage for patients with GC, an effect that was especially pronounced for *PSME3* and *PSME4* (Figure 2B). Additionally, the expression levels of all *PSME* family genes were significantly associated with historical subtypes, nodal metastasis status, and *H. pylori* infection status for patients with GC (Figures 2C–E).

### Diagnostic and Prognostic Significance of Expression Levels of *PSME* Genes in Gastric Cancer Patients

**FIGURE 3:**
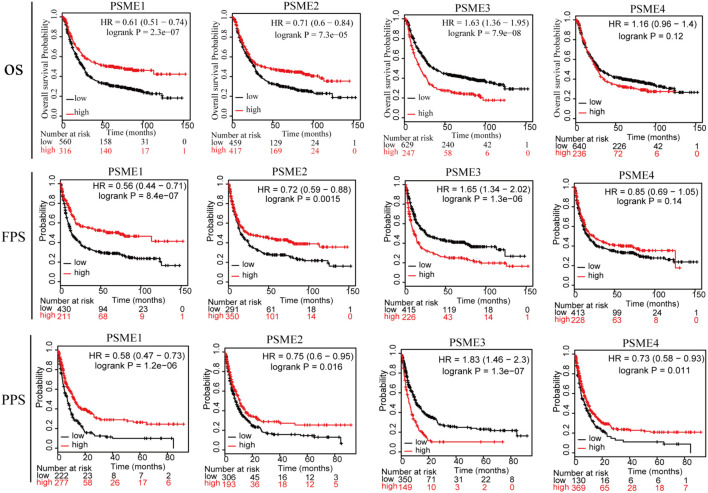
The relationship between the expression levels of the PSME genes and prognosis of GC patients (Kaplan–Meier plotter database). PSME, proteasome activator subunit; OS, overall survival; FPS, first progression survival; and PPS, post-progression survival. Logrank *P* was set at 0.05.

In order to assess the diagnostic significance of *PSME* family genes for distinguishing patients with GC from healthy individuals, we conducted ROC curve using data from TCGA database. The results indicated that PSME3 and PSME4 had high diagnostic performance for distinguishing patients with GC from healthy individuals (the AUC value for *PSME3* and *PSME4* was 0.808 and 0.821, respectively), while PSME1 and PSME2 had moderate performance (the AUC value for *PSME1* and *PSME2* was 0.557 and 0.452, respectively; Supplementary Figure 3). Furthermore, we estimated the OS, FPS, and PPS of GC patients using the Kaplan–Meier method and compared the results using the logrank test to evaluate the relationship between the expression levels of the *PSME* genes and prognosis of GC patients. All *PSME* family genes were significantly correlated with prognosis in patients with GC (Figure 3). The upregulation of *PSME1* and *PSME2* was positively correlated with better prognosis, indicated by longer OS, FPS, and PPS, in GC patients. *PSME4* was strongly associated with favorable PPS. However, the upregulation of PSME3 was significantly related to unfavorable prognosis in GC patients.

### Association Between Prognostic Significance of *PSME* Genes in Gastric Cancer Patients With Diverse Clinicopathological Features

In order to clarify the potential prognostic significance of *PSME* genes in patients with GC, we analyzed the relationship between expression levels of *PSME* genes and survival time of GC patients with different clinicopathological features, such as gender, Lauren classification, differentiation, HER2 status, and different kinds of treatment. Kaplan–Meier curve with log rank test analyses indicated that a high expression of *PSME1* and *PSME2* was significantly related with favorable OS in male and female GC patients, HER2-positive and HER2-negative GC patients, GC patients classified in all Lauren categories, and GC patients with surgery alone. Nevertheless, the upregulation of *PSME3* was negatively correlated with better OS in male and female GC patients, both HER2-positive and HER2-negative, intestinal and mixed classification, poor and well differentiation, and 5-FU-based adjuvant treatment. Finally, the upregulation of *PSME4* was significantly correlated with worse OS in HER2-negative patients and in GC patients with moderately and well-differentiated tumor cells (Table 1).

**TABLE 1:** The correlation between PSME genes and OS in different subtypes of GC patients (Kaplan–Meier plotter).

			PSME1		PSME2		PSME3		PSME4	
Subtypes		Cases	HR (95% CI)	*P*-value	HR (95% CI)	*P*-value	HR (95% CI)	*P*-value	HR (95% CI)	*P*-value
Gender	Male	545	0.63 (0.5–0.79)	**<0.001**	0.68 (0.54–0.86)	**0.001**	2.04 (1.55–2.69)	**<0.001**	0.79 (0.63–1.01)	0.055
	Female	236	0.59 (0.42–0.85)	**0.004**	0.56 (0.39–0.8)	**0.001**	1.59 (1.09–2.32)	**0.014**	1.44 (0.99–2.09)	0.056
HER2	Positive	344	0.66 (0.49–0.89)	**0.007**	1.25 (0.96–1.64)	0.102	1.54 (1.15–2.05)	**0.003**	0.79 (0.61–1.03)	0.077
	Negative	532	0.56 (0.44–0.72)	**<0.001**	0.52 (0.41–0.66)	**<0.001**	1.65 (1.26–2.16)	**<0.001**	1.35 (1.06–1.72)	**0.017**
Lauren classification	Intestinal	320	0.55 (0.39–0.77)	**0.001**	0.46 (0.33–0.64)	**<0.001**	3.03 (1.92–4.75)	**<0.001**	0.74 (0.52–1.05)	0.094
	Diffuse	241	0.53 (0.36–0.79)	**0.002**	0.51 (0.36–0.72)	**<0.001**	1.27 (0.87–1.87)	0.218	1.21 (0.86–1.69)	0.279
	Mixed	32	0.09 (0.01–0.66)	**0.007**	0.15 (0.02–1.17)	**0.038**	2.99 (1.04–8.58)	**0.032**	4.53 (0.59–34.45)	0.110
Differentiation	Poor	165	0.79 (0.53–1.18)	0.249	0.67 (0.45–1)	**0.050**	1.54 (1.01–2.33)	**0.042**	0.81 (0.54–1.2)	0.292
	Moderate	67	0.48 (0.2–1.12)	0.083	0.49 (0.24–1.01)	**0.049**	1.58 (0.79–3.17)	0.194	2.05 (1–4.23)	**0.046**
	Well	32	0.43 (0.18–1.01)	**0.047**	1.41 (0.59–3.36)	0.431	10.5 (1.4–78.81)	**0.005**	2.73 (0.99–7.5)	**0.043**
Treatment	Surgery alone	382	0.46 (0.32–0.65)	**1E-05**	0.48 (0.35–0.67)	**8.1E-06**	0.83 (0.62–1.12)	**0.033**	1.34 (0.97–1.84)	0.073
	5-FU-based adjuvant	153	1.48 (1.04–2.11)	**0.029**	1.98 (1.33–2.96)	**0.001**	1.72 (1.18–2.51)	**0.004**	1.37 (0.9–2.08)	0.136
	Others	76	0.4 (0.16–0.99)	**0.041**	0.28 (0.11–0.71)	**0.004**	0.61 (0.23–1.59)	0.307	1.48 (0.59–3.71)	0.401

*Notes: The P-value was set at 0.05, and the bold values indicate that the results are statistically significant. Abbreviations: PSME, proteasome activator subunit; OS, overall survival; HR, hazard ratio; and CI, confidence interval.*

High expression levels of *PSME1* and *PSME2* were positively related with longer FPS in both male and female, both HER2-positive and HER2-negative, and all Lauren classification patients, while a high expression level of *PSME3* was related with shorter FPS. Additionally, the up-regulated expression of *PSME1* and *PSME2* was associated with favorable FPS in GC patients with surgery-alone treatment, while *PSME4* indicated unfavorable FPS. Besides, the upregulation of *PSME2* and *PSME3* was significantly associated with poor FPS in patients with 5-FU-based adjuvant treatment (Table 2). The up-regulated expression levels of *PSME1* and *PSME2* were significantly associated with favorable PPS in female GC patients, while up-regulated *PSME3* was associated with unfavorable PPS in male patients. A high expression of *PSME1*, *PSME2*, and *PSME4* was positively related with longer PPS in patients with both HER2-negative and HER2-positve status, but high *PSME3* expression levels indicated poor PPS in these patients. The upregulation of PSME1 and PSME2 was positively associated with better PPS in patients with intestinal and diffuse classification, while PSME3 was related with poor PPS in intestinal patients. Meanwhile, PSME1 and PSME2 would be a favorable promising factor in surgery-alone and other treatments, while significantly related with poor PPS in 5-FU-based adjuvant treatment (Table 3).

**TABLE 2:** The correlation between PSME genes and FPS in different subtypes of GC patients (Kaplan–Meier plotter).

			PSME1		PSME2		PSME3		PSME4	
Subtypes		Cases	HR (95% CI)	*P*-value	HR (95% CI)	*P*-value	HR (95% CI)	*P*-value	HR (95% CI)	*P*-value
Gender	Female	201	0.53 (0.35–0.8)	**0.002**	0.6 (0.41–0.88)	**0.008**	1.47 (1–2.16)	**0.047**	1.24 (0.84–1.83)	0.280
	Male	438	0.59 (0.46–0.75)	**<0.001**	0.72 (0.56–0.92)	**0.008**	1.99 (1.48–2.67)	**<0.001**	0.78 (0.6–1.01)	0.056
HER2	Positive	233	0.62 (0.44–0.87)	**0.005**	1.23 (0.89–1.7)	0.215	2.02 (1.43–2.86)	**<0.001**	0.64 (0.45–0.92)	**0.016**
	Negative	408	0.50 (0.45–0.76)	**<0.001**	0.55 (0.43–0.72)	**<0.001**	1.61 (1.2–2.15)	**0.001**	1.26 (0.94–1.68)	0.121
Lauren classification	Intestinal	263	0.52 (0.36–0.74)	**0.002**	0.44 (0.31–0.63)	**<0.001**	2.47 (1.55–3.96)	**<0.001**	0.71 (0.49–1.01)	0.055
	Diffuse	241	0.53 (0.36–0.79)	**0.001**	0.51 (0.36–0.72)	**<0.001**	1.27 (0.87–1.87)	0.218	1.21 (0.86–1.69)	0.280
	Mixed	32	0.09 (0.01–0.66)	**0.003**	0.15 (0.02–1.17)	**0.038**	2.99 (1.04–8.58)	**0.032**	4.53 (0.59–34.45)	0.150
Differentiation	Poor	121	0.7 (0.44–1.14)	0.144	0.71 (0.45–1.14)	0.154	1.78 (1.06–2.98)	**0.027**	0.72 (0.45–1.17)	0.182
	Moderate	67	1.79 (0.94–3.38)	0.071	0.49 (0.24–0.97)	**0.037**	1.34 (0.68–2.66)	0.393	2.18 (1.09–4.35)	**0.023**
Treatment	Surgery alone	382	0.47 (0.34–0.67)	**<0.001**	0.48 (0.35–0.66)	**<0.001**	0.79 (0.6–1.06)	0.112	1.44 (1.06–1.97)	**0.020**
	5-FU-based adjuvant	153	1.25 (0.88–1.78)	0.214	1.67 (1.14–2.46)	**0.008**	1.83 (1.27–2.64)	**0.001**	1.42 (0.97–2.07)	0.070
	Others	80	0.52 (0.23–1.16)	0.103	0.35 (0.16–0.78)	**0.007**	1.53 (0.69–3.36)	0.289	1.53 (0.68–3.48)	0.301

*Notes: The P-value was set at 0.05, and the bold values indicate that the results are statistically significant. Abbreviations: PSME, proteasome activator subunit; FPS, first progression survival; HR, hazard ratio; and CI, confidence interval.*

**TABLE 3:** The correlation between PSME genes and PPS in different subtypes of GC patients (Kaplan–Meier plotter).

			PSME1		PSME2		PSME3		PSME4	
Subtypes		Cases	HR (95%CI)	*P*-value	HR (95%CI)	*P*-value	HR (95%CI)	*P*-value	HR (95%CI)	*P*-value
Gender	Female	149	0.55 (0.36–0.85)	**0.006**	0.47 (0.3–0.74)	**0.001**	1.47 (0.95–2.28)	0.081	1.35 (0.88–2.06)	0.165
	Male	349	0.59 (0.46–0.71)	**<0.001**	0.78 (0.6–1.03)	0.076	2.23 (1.63–3.04)	**<0.001**	0.62 (0.46–0.83)	**0.001**
HER2	Positive	165	0.66 (0.47–0.94)	**0.022**	1.59 (1.11–2.27)	**0.010**	2.33 (1.6–3.4)	**<0.001**	0.54 (0.34–0.84)	**0.005**
	Negative	334	0.53 (0.4–0.7)	**<0.001**	0.54 (0.4–0.71)	**<0.001**	1.64 (1.21–2.23)	**0.001**	0.72 (0.52–0.99)	**0.039**
Lauren classification	Intestinal	192	0.52 (0.34–0.79)	**0.002**	0.48 (0.29–0.79)	**0.003**	3.24 (1.96–5.38)	**<0.001**	0.52 (0.34–0.8)	**0.003**
	Diffuse	176	0.51 (0.35–0.76)	**0.001**	0.46 (0.31–0.67)	**<0.001**	0.73 (0.48–1.11)	0.145	1.19 (0.81–1.76)	0.380
Differentiation	Poor	49	0.65 (0.33–1.27)	0.204	1.68 (0.85–3.35)	0.134	2.47 (1.17–5.23)	**0.015**	1.68 (0.73–3.86)	0.220
	Moderate	24	1.5 (0.57–3.92)	0.406	1.76 (0.51–6.14)	0.366	5.15 (1.66–15.93)	**0.002**	0.42 (0.16–1.15)	0.083
Treatment	Surgery alone	277	0.53 (0.39–0.73)	**<0.001**	0.57 (0.42–0.78)	**<0.001**	1.48 (1.06–2.07)	**0.019**	1.26 (0.9–1.77)	0.174
	5-FU-based adjuvant	136	1.63 (1.12–2.35)	**0.009**	1.78 (1.17–2.7)	**0.006**	1.4 (0.95–2.06)	0.086	0.77 (0.52–1.13)	0.176
	Others	74	0.3 (0.11–0.81)	**0.012**	0.29 (0.11–0.73)	**0.005**	0.48 (0.18–1.26)	0.126	1.67 (0.64–4.38)	0.294

*Notes: The P-value was set at 0.05, and the bold values indicate that the results are statistically significant. Abbreviations: PSME, proteasome activator subunit; PPS, post progression survival; HR, hazard ratio; and CI, confidence interval.*

**FIGURE 4:**
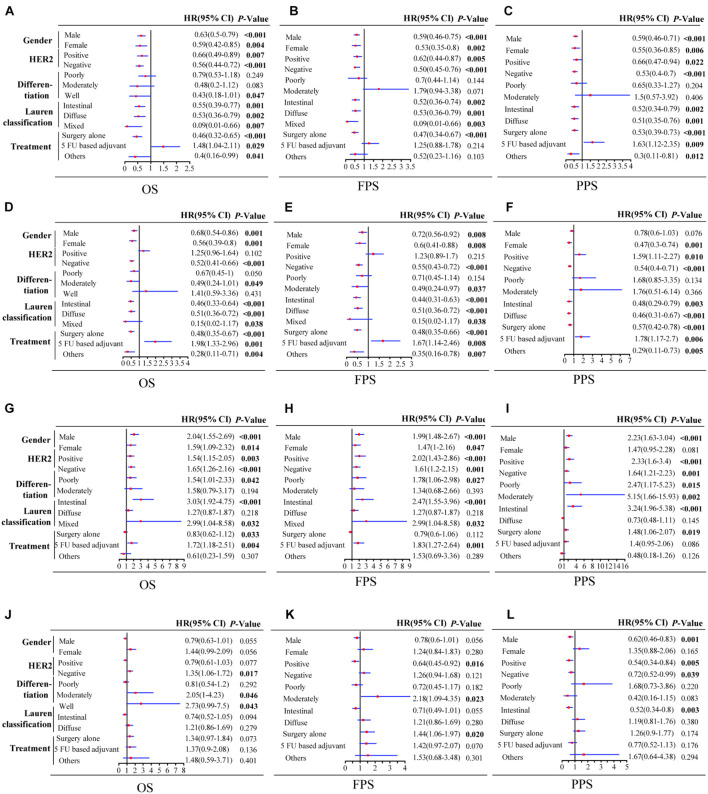
Forest plot of individual expression level of PSME family genes with OS, FPS, and PPS in different clinicopathological feature patients with GC. PSME1 **(A–C)**; PSME2 **(D–F)**; PSME3 **(G–I)**; and PSME4 **(J–L)**. The *P*-value was set at 0.05, and the bold values indicate that the results are statistically significant. OS, overall survival; FPS, first progression survival; PPS, post-progression survival; HR, hazard ratio; and CI, confidence interval.

Taken together, these results demonstrate that a high expression of *PSME1* and *PSME2* is positively related with favorable OS, FPS, and PPS in GC patients with most clinicopathological features, while high expression levels of these genes are significantly correlated with poor prognosis in GC patients with 5-FU-based adjuvant treatment. On the contrary, the upregulation of *PSME3* was significantly related with unfavorable prognosis in GC patients with most clinicopathological features. Finally, the upregulation of *PSME4* indicated poor OS and FPS in GC patients with moderate differentiation (Figure 4).

### Genetic Alterations in *PSME* Genes in Gastric Cancer Patients

**FIGURE 5:**
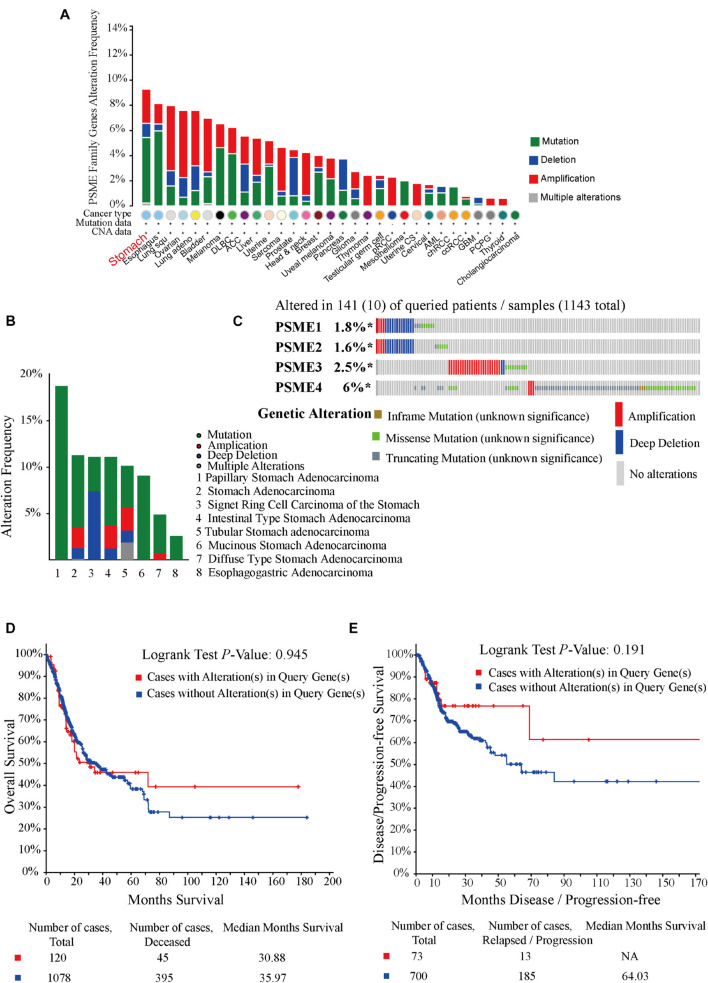
Oncoprint and alteration differences of PSME genes in gastric cancer. **(A)** Summary of genetic alteration in PSME genes across all cancer types. **(B)** Alteration frequency plot of the PSME genes in gastric cancer with different histological subtypes. **(C)** The visual summary oncoprint based on a query of the PSME genes in gastric cancer. **(D)** Kaplan–Meier plots comparing OS in cases with and without PSME family gene alterations. **(E)** Kaplan–Meier plots comparing disease/progression-free survival (DFS/PFS) in cases with and without PSME gene alterations.

In order to investigate the potential roles of *PSME* genes in patients with GC, genetic alterations in these genes were analyzed based on TC3A, GSCALite, and cBioPortal databases. The results showed that *PSME* family gene alteration frequency was the highest in stomach cancer across all tumor types (Figure 5A). Genetic alterations of the *PSME* family genes involved single-nucleotide polymorphism (SNPs), insertion, and deletions (Supplementary Figure 4). mRNA mutations were the most important factor for alterations in different subtypes of GC (Figure 5B). *PSME* genes were altered in 10% (141/1,433) of the GC patients analyzed (Figure 5C). The percentages of genetic alterations in *PSME* genes for GC varied from 1.6 to 6% for individual genes (*PSME1*, 1.8%; *PSME2*, 1.6%; *PSME3*, 2.5%; and *PSME4*, 6%). However, there was no statistical difference in OS and disease-free survival in cases with and without *PSME* genes altered (Figures 5D,E). Then, we further investigated the role of the PSME family genes in drug sensitivity and found that low *PSME4* expression levels conferred resistance to 23 drugs or small molecules, and low *PSME1* levels conferred resistance to 17 drugs or small molecules (Supplementary Figure 5).

### Effects of Mutations in *PSME* Genes

**FIGURE 6:**
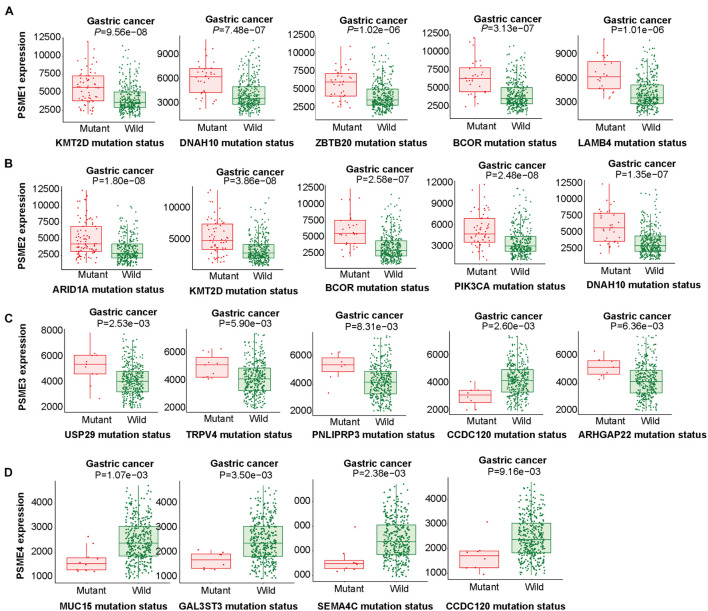
Linking expression changes to mutations. The top five genes whose mutations are most strongly associated with PSME family gene expression changes in GC. **(A–D)** The top five genes whose mutations are most strongly associated with PSME1, PSME2, PSME3, and PSME4, respectively, expression changes in GC.

The abovementioned results revealed that mRNA mutation of *PSME* genes were the most important factor for genetic alterations in GC. Thus, we further analyzed dysregulated genes that are significantly associated with mutations in members of the *PSME* and pinpoint mutations correlated with differential gene expression of *PSME* genes. According to our results, *PSME1* expression was increased in GC patients with mutations in *KMT2D*, *DNAH10*, *ZBTB20*, *BCOR*, and *LAMB4* (Figure 6A). *PSME2* expression was increased in GC patients with mutations in *ARID1A*, *KMT2D*, *BCOR*, *PIK3CA*, and *DNAH10* (Figure 6B). *PSME3* expression was up-regulated in *USP29*, *TRPV4*, *PNLPRP3*, and *ARHGAP22* mutated tumors and down-regulated in patients with *CCDC120* mutations (Figure 6C). Finally, *PSME4* expression was decreased in patients with mutations in *MUC15*, *GAL3ST3*, *SEMA4C*, and *CCDC120* (Figure 6D). Additionally, our results further revealed most of these genes are significantly dysregulated in GC tissues, and the expression of these genes significantly correlated with OS in patients with GC (Supplementary Figure 6). Finally, we identified the top five genes with the strongest dysregulation that were significantly correlated with *PSME* gene mutations (Supplementary Figure 7).

### N6-Methyladenosine Modification Analysis

**FIGURE 7:**
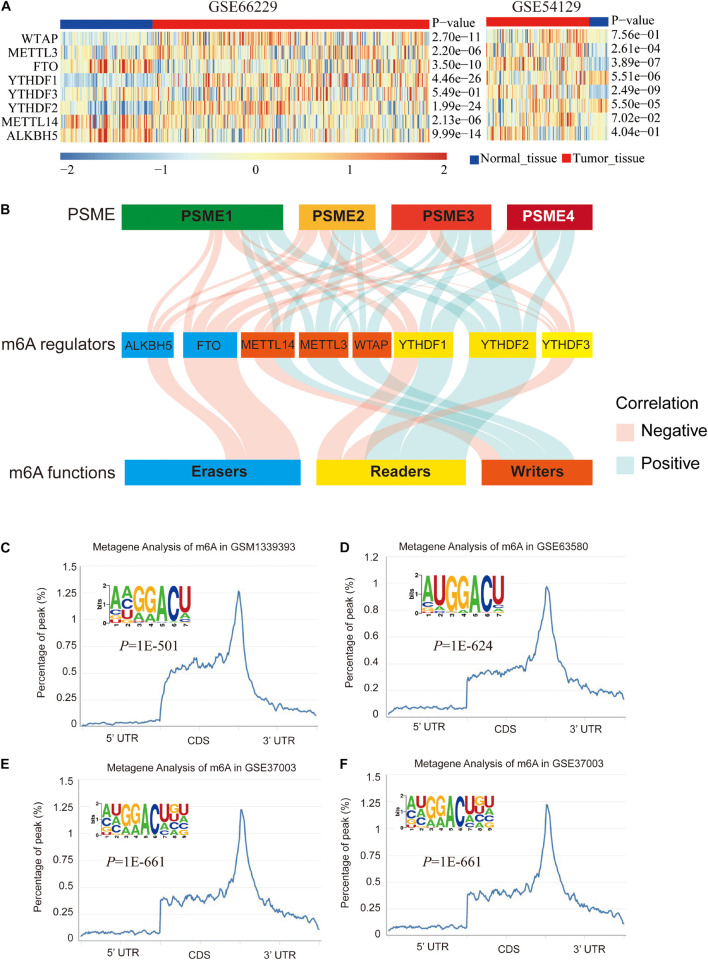
m6A modification of PSME genes. **(A)** The expression levels of m6A-related genes in GSE66229 and GSE54129 datasets. **(B)** Alluvial diagram showed the correlation between m6A-related gene expression and PSME genes. **(C–F)** The normalized distribution of m6A peaks and identified m6A motif in PSME1–4, respectively, based on RMBase V2.0 database.

We explored the epigenetic modification of *PSME* family genes and found that N6-methyladenosine (m6A) was the most frequent modification (Supplementary Table 4). m6A has been functionally characterized as the most abundant internal epigenetic modification and influences the translation, stability, and splicing of mRNAs (Wang et al., 2015; Deng et al., 2018). Recent studies have uncovered that m6A modification plays essential roles in numerous types of cancers, including colorectal cancer (Chen et al., 2020; Hu et al., 2020), GC (Yue et al., 2019), glioma (Tu et al., 2020), hepatocellular carcinoma (Xu et al., 2020), and bladder cancer (Han et al., 2019). Therefore, we further analyzed m6A modification in the members of the *PSME* family. Expression levels of m6A-related genes were significantly dysregulated in GC tissues (Figure 7A), and *PSME* family gene expression levels were significantly correlated with the expression of most of these genes (Figure 7B and Supplementary Figures 8A–H). We validated the results in the TCGA-STAD dataset using the TIMER (Supplementary Figure 8I). The m6A consensus motif of *PSME1–4* is shown in Figures 7C–F. We found a similar m6A distribution pattern in which m6A peaks were enriched in CDS and 3′UTR with a steep density peak around the stop codon.

### Functional Enrichment Analysis of *PSME* Genes in Gastric Cancer Patients

**FIGURE 8:**
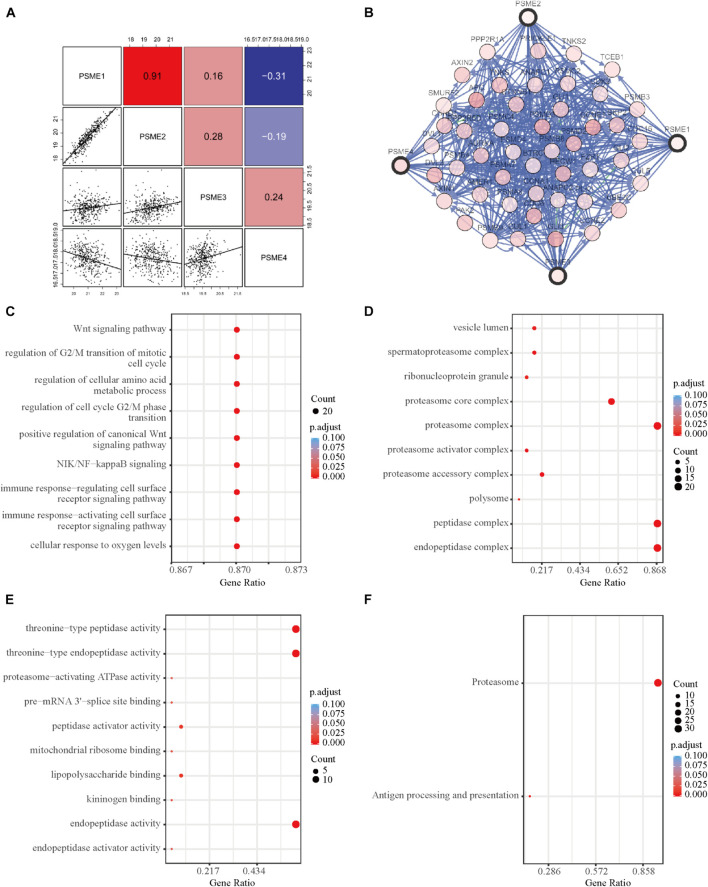
Functional enrichment and correlation analysis of PSME genes in patients with GC. **(A)** Pearson correlation analysis of individual among PSME genes. **(B)** The network for PSME genes and the most frequently altered neighbor genes using TC3A database. **(C)** Biological process analysis. **(D)** Cellular components. **(E)** Molecular function. **(F)** Kyoto Encyclopedia of Genes and Genomes (KEGG) analysis. All of terms colored by adjusted *P*-value, and the size of points represent number of genes.

In order to further explore the potential molecular mechanisms underlying the dysregulation of the expression levels of *PSME* genes, we assessed the correlation between the individuals’ expression levels using the Pearson correlation coefficient. *PSME1* and *PSME2* had a strong positive correlation, while *PSME1* and *PSME4* and *PSME2* and *PSME4* were negatively correlated (Figure 8A). Additionally, a PPI network was created using the TC3A database and GO term and KEGG enrichment analysis was performed. The PPI network revealed that *TCEB1*, *PSMB3*, *CCNE2*, *BTRC*, and *AMER1* were closely associated with alterations in *PSME* family genes (Figure 8B). GO term and KEGG pathway enrichment analysis indicated that members of the PSME family and their most frequently altered neighbor genes were mainly involved in the Wnt signaling pathway, NIK/NF-κB pathway, cell cycle regulation, cellular response to oxygen levels, immune response, and proteasome activity, which are important tumor-related processes (Figures 8C–F).

### Gene Set Enrichment Analysis

**FIGURE 9:**
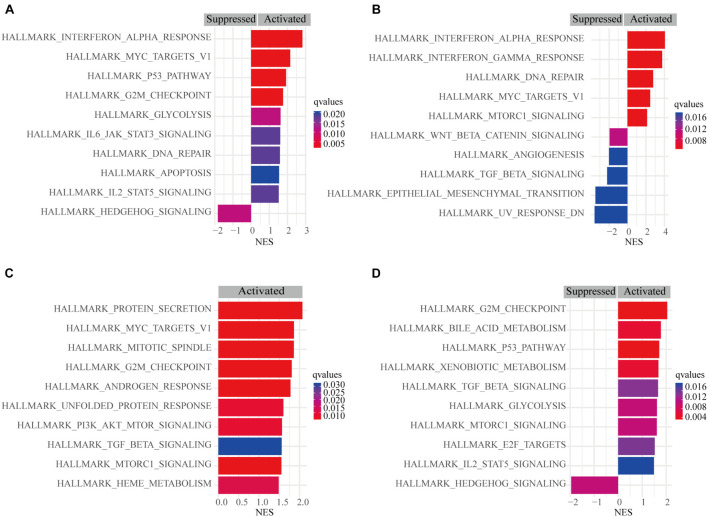
Gene set enrichment analysis (GSEA) of PSME genes in GC. **(A)** PSME1. **(B)** PSME2. **(C)** PSME3. **(D)** PSME4. NES, normalized enrichment score. Only the 10 most common functional pathways enriched were listed. *q*-values were adjusted *P*-values and was set at 0.05.

A pathway-based analysis in GC showed that *PSME1* mainly activated the interferon-alpha response, P53 pathway, G2M checkpoint, and DNA repair, while it suppressed hedgehog signaling pathway in GC tumors (Figure 9A). *PSME2* activated interferon-alpha response, DNA repair, and mTORC1 signaling pathway and suppressed the Wnt/β-catenin and TGF-β signaling pathway in GC patients (Figure 9B). *PSME3* mainly activated PI3K-AKT-mTORC1 signaling pathway, G2M checkpoint, and TGF-β signaling pathway in GC patients (Figure 9C). *PSME4* mainly activated G2M checkpoint and the P53 signaling and TGF-β pathway, while suppressed hedgehog signaling pathway in GC patients (Figure 9D). Additionally, we analyzed the target genes of members of the *PSME* family that were present in the Reactome pathway and Kinase Target databases using the LinkedOmics database (Supplementary Figures 9, 10). Consistent with the above analysis, *PSME* family genes were mainly involved in cell cycle; interferon-alpha response; DNA repair; the P53, TGF-β, and Wnt signaling pathways; and immune-related signaling pathways, all of which are involved in the biology of cancer.

### Immune Infiltration Is Associated With *PSME* Genes in Gastric Cancer

**FIGURE 10:**
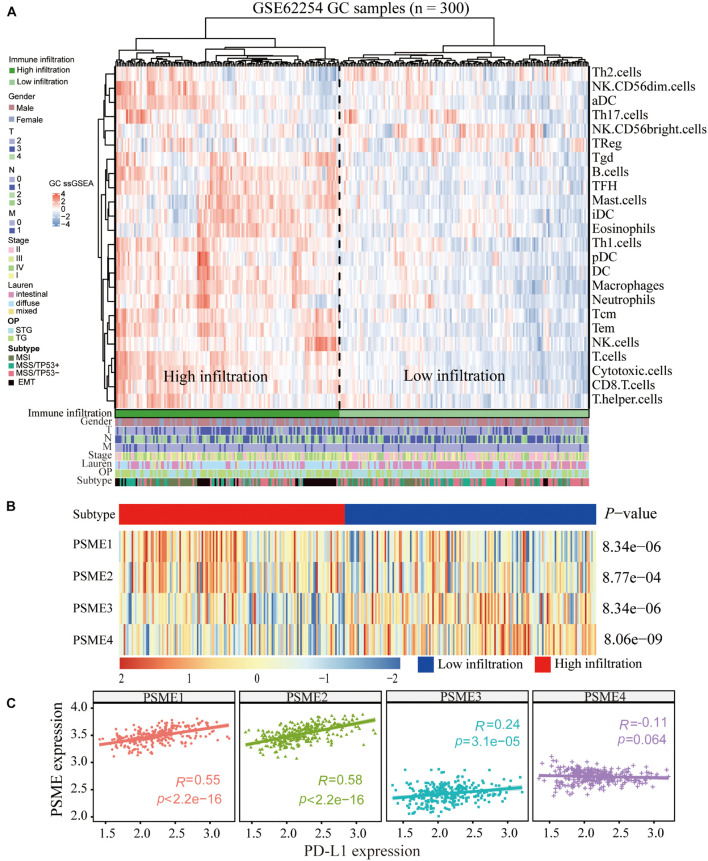
Correlation between PSME genes and immune infiltration. **(A)** Immune landscape of GC and unsupervised clustering of 300 patients from the GSE62254 cohort. Molecular subtype, post-operation type, number of positive nodes, Lauren classification, stage, TNM stage, age, and gender were annotated in the higher panel. Hierarchical clustering was performed with Euclidean distance and Ward linkage. **(B)** The relative expression of PSME genes in higher and lower immune infiltration patients with GC. **(C)** The correlation between PSME genes’ expression and PD-L1 in GC patients in GSE62254 cohort.

Although the complex interactions between solid tumors and their microenvironment remain unclear, numerous studies have shown that the infiltration level of immune cells is strongly correlated with the progression and prognosis of GC (Bindea et al., 2013; Pan et al., 2019; Xiao et al., 2020). Based on the expression data extracted from GSE62254, we applied the ssGSEA deconvolution algorithm to determine the relative abundance of each immune cell type (Figure 10A). Interestingly, *PSME* genes were differentially expressed in the higher and lower immune infiltration groups (Figure 10B). *PSME1* and *PSME2* were highly expressed in the high-infiltration group, while *PSME3* and *PSME4* were down-regulated in the high-infiltration group. Furthermore, *PSME* genes were strongly related to the infiltration of immune cells (Supplementary Figure 11) and significantly correlated with the most immune-related genes (Supplementary Figure 12), indicating that *PSME* genes play an essential role in GC partially due to their effect on immune infiltration.

The above analysis confirmed that *PSME* gene expression was related to immune infiltration in GC and significantly correlated with prognosis. Therefore, we hypothesized that the expression level of *PSME* genes may influence the prognosis of GC patients partially due to their impact on immune infiltration. Hence, we conducted a prognostic analysis based on the expression levels of *PSME* genes of GC in the related immune cell subgroup using the Kaplan–Meier plotter database. The findings indicated that *PSME* gene expression levels may affect prognosis, partly because of immune infiltration in GC (Supplementary Figures 13A–D).

### The Role of *PSME* Genes in the Prediction of Immunotherapeutic Benefits

In summary, the above results indicate that *PSME* genes, especially *PSME1* and *PSME2*, can act as potential biomarkers for immunotherapy. Therefore, we analyzed the correlation between the expression levels of members of the *PSME* and *PD-L1* and found that expression levels of *PSME1–3* were significantly positively correlated with *PD-L1* expression levels in GSE62254 (Figure 10C). Furthermore, we analyzed the correlation between *PSME* genes and the expression of immune checkpoint-relevant genes, including *PD-L1*, *PD-1*, *LAG3*, and *CTLA4*, in 33 cancer types in TCGA database (Supplementary Figure 14). The results showed that *PSME1* and *PSME2* were positively correlated with the expression levels of four immune checkpoint genes in most types of tumors, including STAD.

**FIGURE 11:**
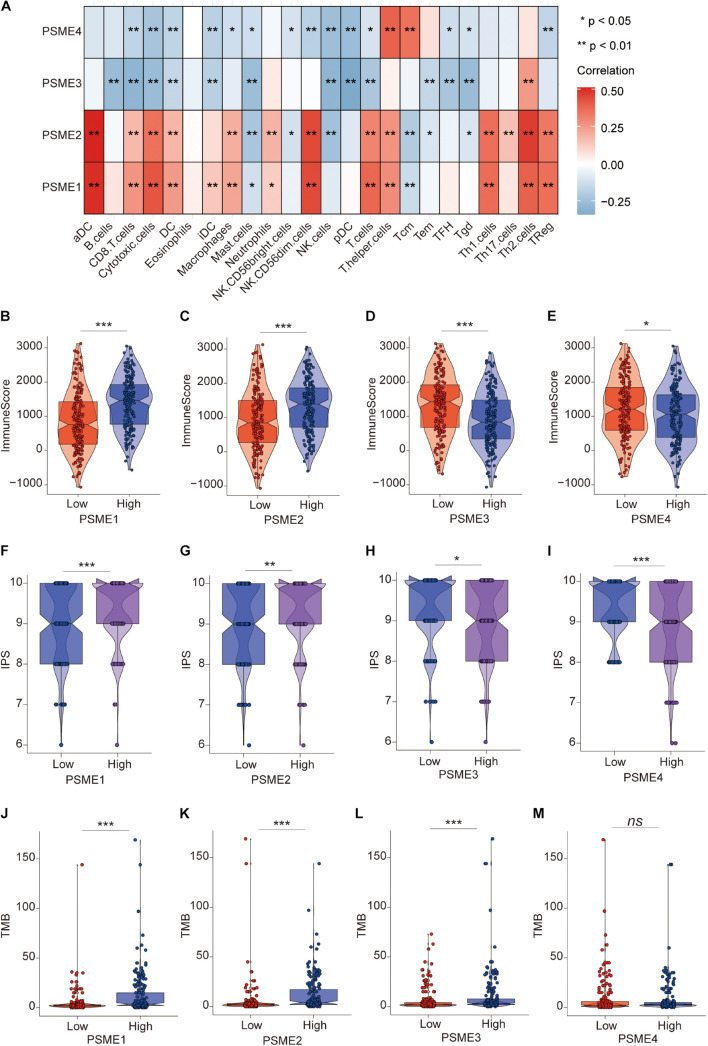
The role of PSME genes in the prediction of immunotherapeutic benefits. **(A)** The correlation of the infiltration levels of immune cells and the expression of PSME genes in TCGA-STAD cohort. *p < 0.05; **p < 0.01; ***p < 0.001; and ****p < 0.0001. **(B–E)** Violin plot depicted the distribution of immunescore in high and low PSME gene expression group [**(B)** PSME1, **(C)** PSME2, **(D)** PSME3, and **(E)** PSME4]. **(F–I)** Violin plot depicted the distribution of IPS in high and low PSME gene expression group [**(F)** PSME1, **(G)** PSME2, **(H)** PSME3, and **(I)** PSME4]. **(J–M)** Violin plot depicted the distribution of tumor mutational burden (TMB) in high and low PSME gene expression group [**(J)** PSME1, **(K)** PSME2, **(L)** PSME3, and **(M)** PSME4].

To verify the above results, we performed ssGSEA again in the TCGA-STAD dataset to analyze the relationship between the expression levels of *PSME* genes and the infiltration level of immune cells. Consistently, *PSME1* and *PSME2* were significantly correlated with the infiltration level of most immune cells, while *PSME3* and *PSME4* were negatively correlated with the infiltration level of most immune cells (Figure 11A). In line with the result, the immune score was significantly higher in the patients with high PSME1 and PSME2 expression than those patients with low expression in STAD (Figures 11B,C). The pattern observed for *PSME3* and *PSME4* expression was the opposite (Figures 11D,E).

Numerous studies have demonstrated that patients with high IPS and tumor mutational burden (TMB) have a better response to immunotherapy. We found that the IPS of patients in the high *PSME1* and *PSME2* expression groups was significantly higher than that in the low *PSME1* and *PSME2* expression groups (Figures 11F,G). In contrast, the IPS of patients in the low *PSME3* and *PSME4* expression groups was significantly higher than those in the high *PSME3* and *PSME4* expression groups (Figures 11H,I). Interestingly, the results of the study showed that the TMB in patients with high *PSME1–3* expression levels was significantly higher than those patients with low *PSME1–3* expression level (Figures 11J–L), while different expression levels of *PSME4* were not associated with statistically different TMB (Figure 11M). In conclusion, these results reveal that PSME genes, especially *PSME1* and *PSME2*, may act as potential biomarkers for response to immunotherapy of GC patients.

### Expression Levels of *PSME* Genes Are Associated With the Anti-cancer Immunity Cycle

**FIGURE 12:**
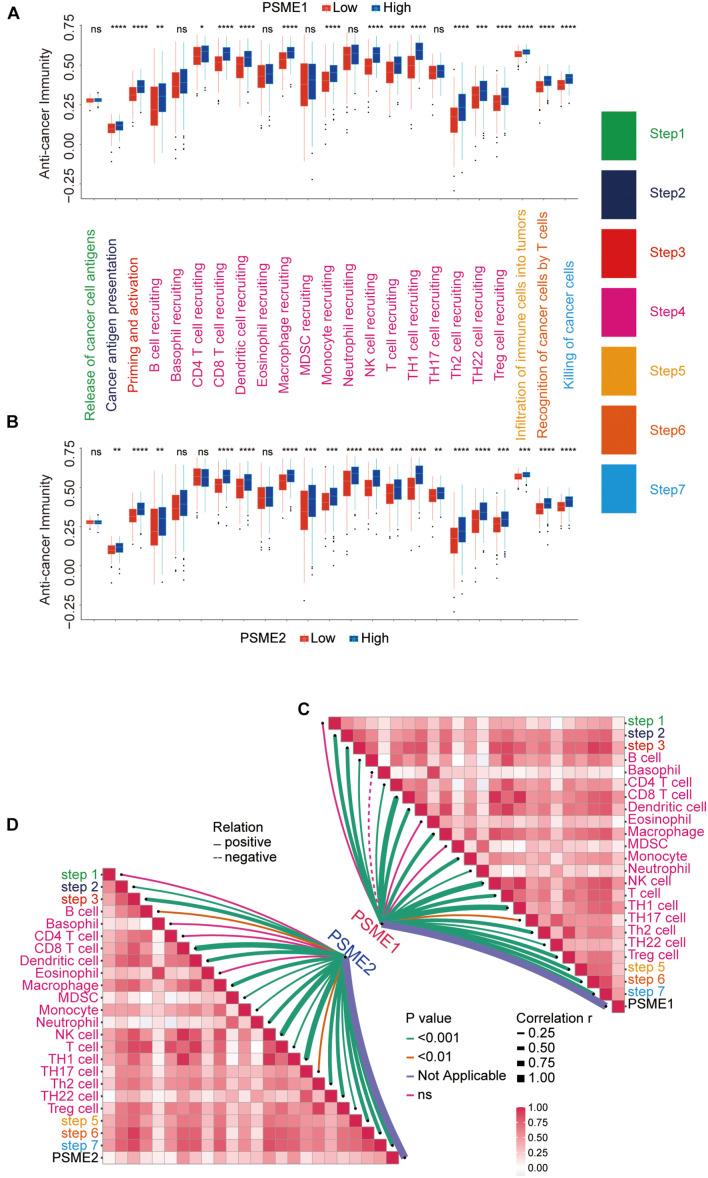
The relationship between PSME genes and anti-cancer immunity cycle. **(A)** Differences in the multiple steps of the anti-cancer immunity cycle in high and low PSME1 groups in GC. **(B)** Differences in the multiple steps of the anti-cancer immunity cycle in high and low PSME2 groups in GC. **(C)** Correlation between PSME1 and the steps of the anti-cancer immunity cycle. **(D)** Correlation between PSME1 and the steps of the anti-cancer immunity cycle. *p < 0.05; **p < 0.01; ***p < 0.001; and ****p < 0.0001.

The success of tumor immunotherapy largely depends on the development and activation of immune cells in the host microenvironment. This comprehensive process would conceptualize the anti-cancer immunity cycle, including the release of cancer antigens (step 1), cancer antigen presentation (step 2), priming and activation (step 3), transfer of immune cells to the tumors (step 4), infiltration of immune cells into the tumors (step 5), recognition of cancer cells by T cells (step 6), and eradication of cancer cells (step 7; Chen and Mellman, 2013; Xu et al., 2018). The state of the seven-step anti-cancer immunity cycle and the level of tumor infiltration of immune cells determine the complex tumor immunophenotype in the tumor microenvironment. Therefore, we further analyzed the relationship between PSME gene expression and the anti-cancer immunity cycle. There was no significant difference in antigen release from tumor cells (step 1) between the groups that had high and low *PSME1* and *PSME2* expression, but cancer antigen presentation (step 2), priming and activation (step 3), trafficking of most immune cells to tumors (step 4), infiltration of immune cells into tumors (step 5), T cell recognition of cancer cells (step 6), and killing of cancer cells (step 7) were significantly higher in patients with high expression of *PSME1/2* than in patients with low expression of *PSME1/2* (Figures 12A,B). However, there was no statistically significant difference in the activation of most of these steps between the high and low expression groups of *PSME3* and *PSME4* (Supplementary Figures 15A,B). We further analyzed the correlation between the expression level of PSME genes and the enrichment score of each step of immunity cycle (Figures 12C,D and Supplementary Figures 15C,D). *PSME1* and *PSME2* were strongly positively correlated with the steps of the immunity cycle.

### Analysis of the Expression Levels of PSME at the Single-Cell Level or Cluster Level in Gastric Cancer

**FIGURE 13:**
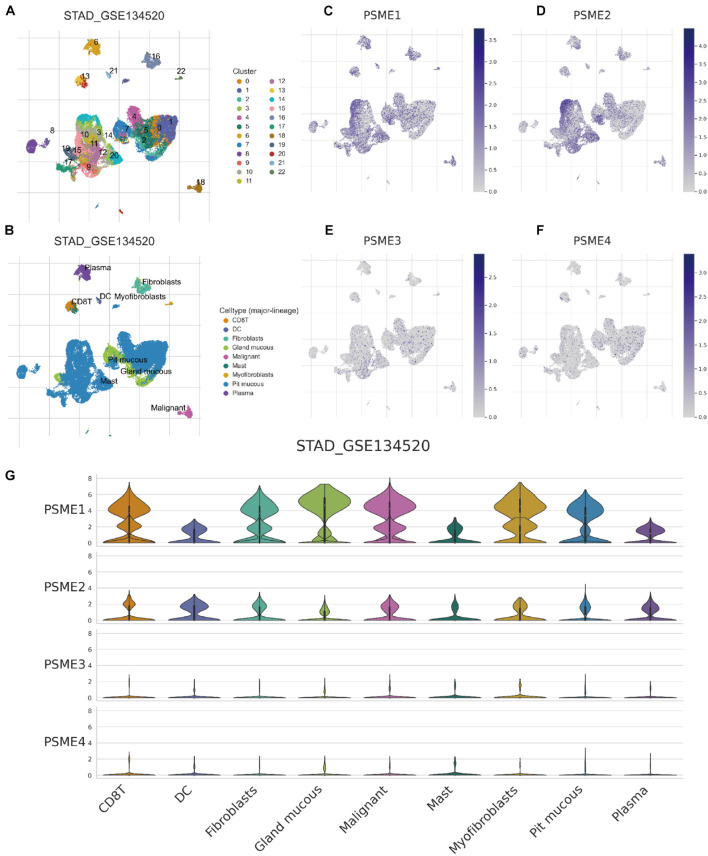
Comparison of PSME family gene expression at single-cell resolution in GC. **(A)** UMAP visualization of dataset STAD_GSE134520. Colors represent the major-lineage cluster ID. **(B)** UMAP visualization of dataset STAD_GSE134520. Colors represent the major-lineage cell-types. **(C–F)** Comparison of PSME gene expression at single-cell resolution in GC. **(G)** The grid violin plot reflects the distribution of PSME gene expression (LogTPM) in different cell types in GC.

Although immunotherapy has become one of the most promising treatment strategies for cancer, only a minority of patients can benefit from immunotherapy because of the random heterogeneity of tumor microenvironment. Recently, scRNA-seq technologies have been adopted to comprehensively characterize immune system heterogeneity in tumor cells. Hence, we characterized gene expression level of members of the PSME family at single-cell resolution based on GSE134520 dataset (Figure 13). Additionally, we analyzed *PSME* gene expression in different cell types derived from different sources, male and female patients, patients with different tumor stages, and patients that have received distinct treatments, respectively, (Supplementary Figures 16, 17). The results indicated that *PSME1* and *PSME2* may be cell-type potential markers (Supplementary Table 5).

## Discussion

Immunotherapy is a promising treatment for cancer. By inhibiting immune checkpoints, tumor-infiltrating immune cells can be activated to attack tumor cells (Helmink et al., 2020). Immunotherapy has been successfully used in multiple cancers, and thousands of patients have benefitted from this treatment (Hugo et al., 2016; Zhao et al., 2019). Despite this success, there is still a large percentage of cancer patients who are resistant to immunotherapy because the complex interactions between solid tumors and their microenvironment remain unclear. Therefore, novel indicators are urgently needed to predict immunotherapy responses. For this purpose, we analyzed the relationship between *PSME* gene expression levels and the expression levels of immune checkpoint-relevant genes (including *PD-L1*, *PD-1*, *CTLA4*, and *LAG3*) and with TMB and IPS.

Previous studies have reported that *PSME1* is overexpressed in multiple cancers, including ovarian cancer (Lemaire et al., 2007; Longuespee et al., 2012), skin cutaneous melanoma (Wang et al., 2019), esophageal squamous cell carcinoma (Zhang et al., 2011), and prostate cancer, suggesting that *PSME1* could be a promising marker and therapeutic target for prostate cancer (Sanchez-Martin et al., 2013). Furthermore, a study recently reported that P*SME1* is significantly up-regulated in OSCC tumor tissues and cell lines and that it is involved in OSCC oncogenesis, with high *PSME1*expression significantly associated with recurrence and worse OS. This study revealed *PSME1* as an independent prognostic predictor in patients with OSCC (Feng et al., 2016). Consistent with previous reports, our findings indicated that PSME1 was up-regulated in GC tumor tissues compared to non-cancerous tissues. Nevertheless, we found that up-regulated *PSME1* was positively associated with favorable OS, FPS, and PPS in GC patients. Additionally, high *PSME1* expression was positively correlated with the infiltration of most immune cells and activation of anti-cancer immunity cycle steps. Moreover, patients with high *PSME1* expression had higher IPS and TMB. The findings indicated that *PSME1* expression level may affect the prognosis of GC patients, partly because it impacts the degree of immune infiltration, and that *PSME1* could act as a potential biomarker for the response to immunotherapy of GC patients.

*PSME2* has been reported to be significantly under-expressed in esophageal squamous cell carcinoma tissues, and up-regulated *PSME2* expression significantly inhibited cell growth, proliferation, and malignancy of tumor cells (Chen J. Y. et al., 2017). In contrast, *PSME2* is up-regulated in endometrial cancer tissues compared to non-cancerous tissues and is closely related to the development of endometrial cancer (Spirina et al., 2012). However, the results from the previous study, in contrast to our findings, indicate that *PSME2* was down-regulated in gastric adenocarcinoma tissues compared to paired normal tissues and regulated GC progression by modulating the expression of chloride intracellular channel 1 (Huang et al., 2010; Zheng et al., 2012). In the present study, we demonstrated that *PSME2* expression was significantly increased in GC tumor tissues compared to non-cancerous tissues. Survival analysis revealed that up-regulated PSME2 expression was positively related to better prognosis, including OS, FPS, and PPS in GC patients. Mechanistically, PSME2 is negatively regulated by the N-α-acetyltransferase 10 protein to regulate multiple pathways related to cancer cell proliferation, apoptosis, and metastasis (Min et al., 2013). We found that *PSME2* activated interferon-alpha response, DNA repair, and MTORC1 signaling pathway, but suppressed Wnt/β-catenin and TGF-β signaling pathways in GC patients. Similar to *PSME1*, our study found that the expression of *PSME2* was positively correlated with the infiltration of most immune cells and the activation of anti-cancer immunity cycle steps. Patients with high *PSME2* expression have higher IPS and TMB. The findings indicated that *PSME2* gene expression level may affect the prognosis and progression of GC patients, partly because of immune infiltration, and *PSME2* may serve as a potential biomarker for GC patients, indicating a response to immunotherapy.

Previous studies have reported that *PSME3* expression is increased in tumor tissues compared to normal tissues and that it acts as an oncogenic driver in many types of cancers (Chai et al., 2015; Li J. et al., 2015; Chen H. et al., 2017). In OSCC tissues, *PSME3* is significantly up-regulated, and high PSME3 expression levels are significantly related to unfavorable prognosis in OSCC patients (Li J. et al., 2015). *PSME3* is significantly overexpressed in colorectal cancer tissue compared with healthy donor tissue, leading to its consideration as a novel serum tumor marker for identifying colorectal cancer patients (Roessler et al., 2006). Similarly, several studies have recently reported that *PSME3* was overexpressed in breast cancer (BRCA) tissues compared to normal tissues and that BRCA patients with low expression levels of *PSME3* had a favorable prognosis compared to patients with higher expression of *PSME3* (Chai et al., 2014, 2015; Shi et al., 2015). Furthermore, *PSME3* plays a crucial role in regulating the cell cycle and inducing epithelial–mesenchymal transition to influence the tumor immune microenvironment in BRCA (Yi et al., 2017). In addition, *PSME3* was significantly up-regulated in pancreatic cancer tissues and cell lines at both the mRNA and protein levels (Yu et al., 2019). Meanwhile, a high expression of *PSME3* was positively correlated with tumor size and negatively correlated with favorable prognosis in patients with pancreatic cancer. It has been reported that *PSME3* plays oncogenic roles in pancreatic cancer by mediating c-Myc degradation to accelerate glycolysis and might act as a new therapeutic target for pancreatic cancer (Guo et al., 2017). The Wnt/β-catenin signaling pathways play an important role in regulating various processes critical to cancer progression, including cell death, tumor growth, tumor initiation, differentiation, and metastasis (Anastas and Moon, 2013; Zhan et al., 2017). Previous studies demonstrated that *PSME3* is necessary in skin tumorigenesis mediated by MAPK/p38 activation of the Wnt/β-catenin signaling pathway (Li L. et al., 2015). Finally, NF-κB signaling pathway is the central coordinator of innate and adaptive immune responses, and *PSME3* enhances the transcriptional activity of the NF-κB pathway to play a crucial role in host defense and innate immunity (Sun J. et al., 2016) and plays a key role in cell growth and apoptosis in GC (DiDonato et al., 2012; Fan et al., 2013). In this report, we demonstrated that *PSME3* is up-regulated in GC tumor tissues compared to normal tissues and that the upregulation of *PSME3* is strongly related to unfavorable OS, FPS, and PPS in GC patients. Furthermore, the expression levels of *PSME3* was negatively correlated with the infiltration of most immune cells, immune score, and IPS. The findings indicate that *PSME3* may play a critical role in GC carcinogenesis, and ROC analysis suggested that PSME3 had high diagnostic performance for distinguishing GC patients from healthy individuals and could serve as a novel diagnostic marker for GC.

*PSME4* plays a vital role in multiple processes, including proteasome assembly (Fehlker et al., 2003), genomic stability (Blickwedehl et al., 2008), and DNA repair (Schmidt et al., 2005). Previous studies reported that *PSME4* plays an indispensable role in the antioxidant response (Huang et al., 2016) and in maintaining glutamine homeostasis, which is particularly important for long-term survival of tumor cells after radiation exposure (Blickwedehl et al., 2012). In the present study, we found that the expression of *PSME4* was higher in GC tumor tissues than in non-cancerous tissues. ROC analysis suggested that *PSME4* had a great diagnostic performance for distinguishing GC patients from healthy individuals and could serve as a good diagnostic marker for GC. Prognostic analysis indicates that the overexpression of *PSME4* is significantly correlated with poor FPS and OS in GC patients with moderate differentiation, while it is related to favorable PPS in GC patients. Mechanistically, our findings indicated that *PSME4* mainly activated the cell cycle, P53 signaling, and TGF-β signaling pathways, while suppressing the hedgehog signaling pathway in GC patients.

The expression of *PSME* genes can be dysregulated in malignancies through various mechanisms, including epigenetic modification, non-coding mutations in promoters or enhancers, and genomic amplification/deletion (Jones et al., 2008; Khurana et al., 2013; D’Antonio et al., 2017; Rheinbay et al., 2017). In the present study, we found that that *PSME* gene alteration frequency was the highest in GC across all tumor types. m6A may be the most frequent modification of to affect the translation and stability of *PSME* family genes. Further analysis indicated that *PSME* genes play a crucial role in GC, which may be partially due to their effect on immune infiltration, and *PSME1–2* may act as potential biomarkers for GC patients, indicating a response to immunotherapy.

The results of this study should be interpreted in the light of its limitations. First, the present study is mostly bioinformatics, and most of these findings result from *in silico* analyses of the data retrieved from public databases and lack verification through *in vitro* and *in vivo* experiments. We verified the mRNA expression of *PSME* genes in 40 GC cases. The protein expression levels of *PSME* genes in GC were explored using the HPA database, but protein expression levels of these genes in GC cells and tumor-infiltrating immune cells were insufficient, and *PSME2* was not found in the HPA database. Besides, the molecular mechanism of *PSME* family genes in GC was investigated, but there was a lack of verification through *in vitro* and *in vivo* experiments. Consequently, the findings of this study still require further verification. Notwithstanding these limitations, to the best of our knowledge, this is the first study to systematically demonstrate the expression levels, prognostic values, mechanism of dysregulation, potential molecular mechanism, and the role in the prediction of immunotherapeutic benefits of *PSME* genes in GC using various large databases and bioinformatics approaches. Our findings may provide new insights for further studies focusing on the underlying mechanisms of *PSME* genes in GC.

## Conclusion

In this study, we systematically demonstrated the expression level prognostic value, the mechanism of dysregulation, potential molecular mechanisms, and the role of these genes in the prediction of immunotherapeutic benefits of *PSME* family genes in GC using various large databases and using an unbiased *in silico* approach. Our findings suggest that *PSME1* and *PSME2* may be potential prognostic markers for enhancing survival and prognostic accuracy in GC patients and may even act as potential biomarkers for GC patients, indicating a response to immunotherapy. *PSME3* may serve as an oncogene in tumorigenesis and may be a promising therapeutic target for GC. *PSME4* had great diagnostic performance for distinguishing GC patients from healthy individuals and could serve as a good diagnostic marker for GC.

## Data Availability Statement

The original contributions presented in the study are included in the article/Supplementary Material, further inquiries can be directed to the corresponding author/s.

## Ethics Statement

Written informed consent was obtained from all patients and the study was approved by the Ethics Committee of the Fourth Affiliated Hospital, Hebei Medical University.

## Author Contributions

YH conceived and designed this study. YG analyzed the data and drafted the manuscript. JJ and XD made the chart and figure. All authors contributed to the article and approved the submitted version.

## Conflict of Interest

The authors declare that the research was conducted in the absence of any commercial or financial relationships that could be construed as a potential conflict of interest.

## Publisher’s Note

All claims expressed in this article are solely those of the authors and do not necessarily represent those of their affiliated organizations, or those of the publisher, the editors and the reviewers. Any product that may be evaluated in this article, or claim that may be made by its manufacturer, is not guaranteed or endorsed by the publisher.

## Footnotes

1
http://xena.ucsc.edu/

2
https://cistrome.shinyapps.io/timer/

3
http://kmplot.com/

4
http://www.cbioportal.org/

5
http://www.mutarget.com/

6
https://david.ncifcrf.gov/

7
http://software.broadinstitute.org/gsea/msigdb/index.jsp

8
http://bioinfo.life.hust.edu.cn/web/GSCALite/

9
http://tisch.comp-genomics.org
