# Supplementary material for: The Expression Patterns and Prognostic Value of the Proteasome Activator Subunit Gene Family in Gastric Cancer Based on Integrated Analysis
Source: Front Cell Dev Biol. 2021 Sep 28;9:663001. doi: 10.3389/fcell.2021.663001 (PMC8505534; doi:10.3389/fcell.2021.663001)
Supplement: Supplementary file 1 [file Data_Sheet_1.docx]

**The expression patterns and prognostic value of the proteasome activator subunit gene family in gastric cancer based on integrated analysis**

**Yongdong Guo^1^, Xiaoping Dong^1^, Jing Jin^1^, Yutong He^1^**

**1 Cancer Institute, Fourth Hospital of Hebei Medical University, Shijiazhuang, 050011, China**

**Correspondence should be addressed to: Yutong He, (E-mail: 15733291685@163.com)**

**Short Title: Proteasome activator subunit genes as predictive gastric cancer markers**

**
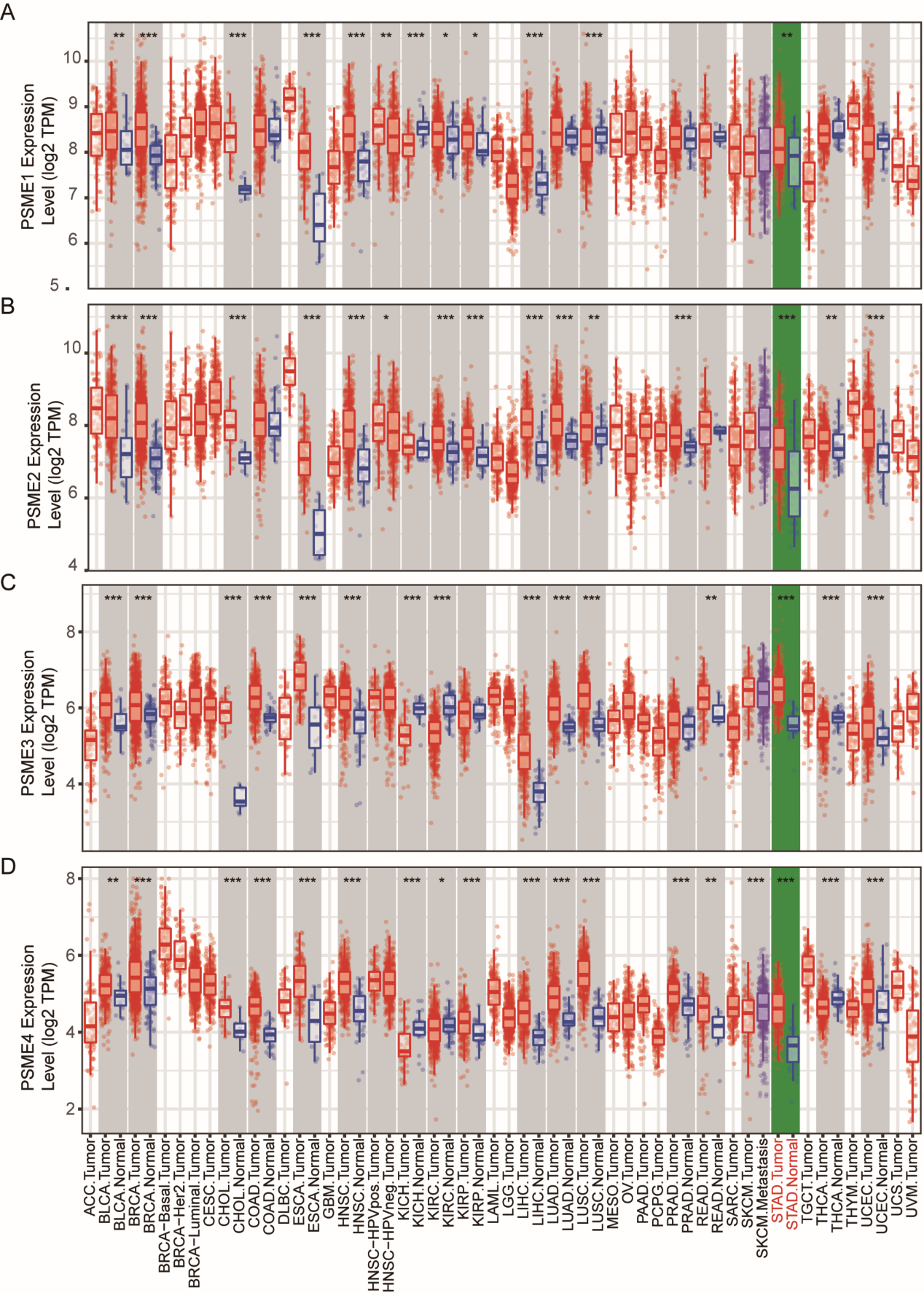
**

**Supplementary Figure 1.** PSME family genes expression across all tumor samples and normal tissues analyzed by TIMER database. *: *P*<0.05, **: *P*<0.01, ***: *P*<0.001, NS: *P* >0.05.


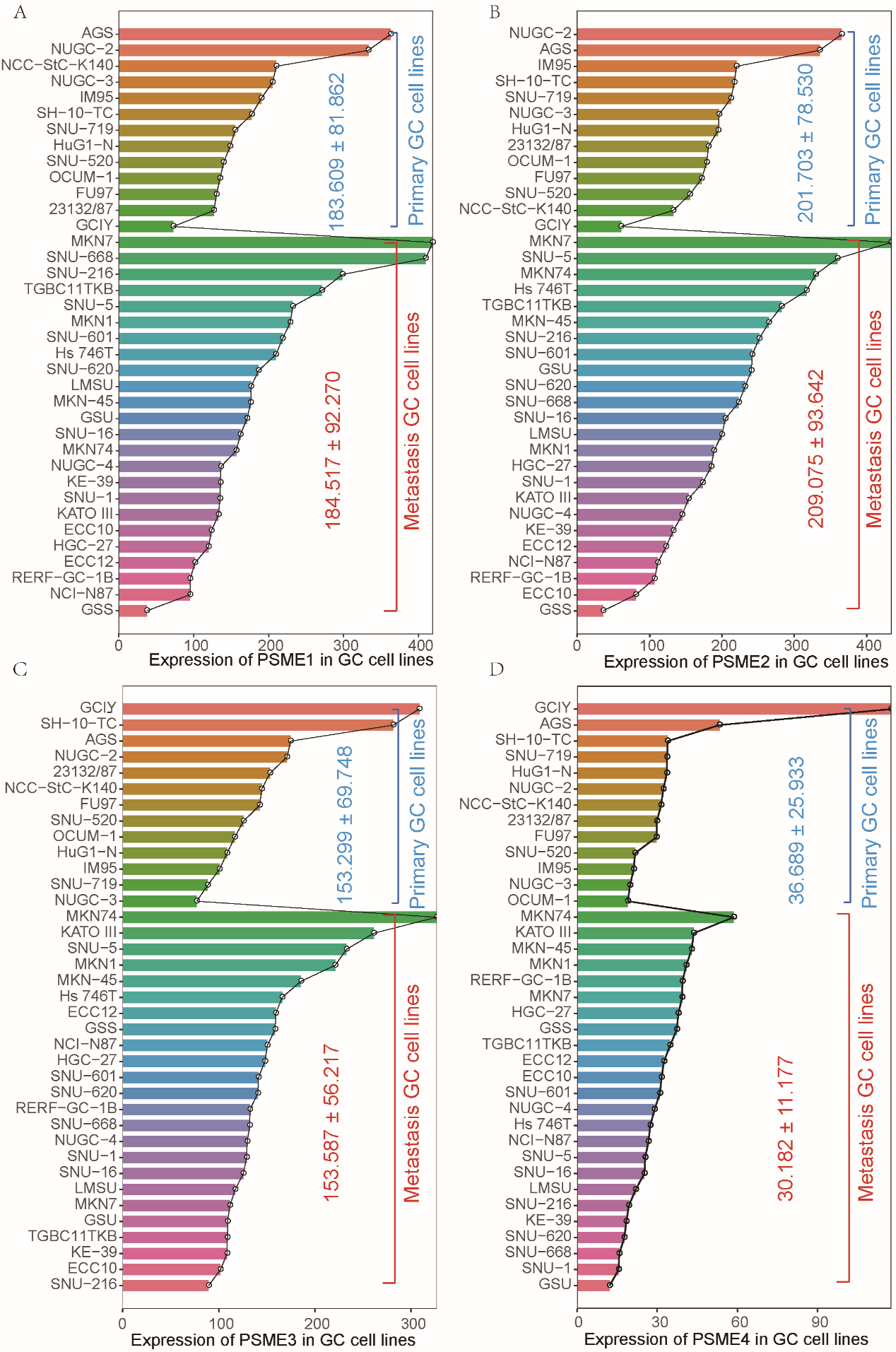


**Supplementary Figure 2.** The expression level of PSME family genes in kinds of GC cell lines using the data obtained from CCLE database. A: PSME1, B: PSME2, C: PSME3, D: PSME4.

**
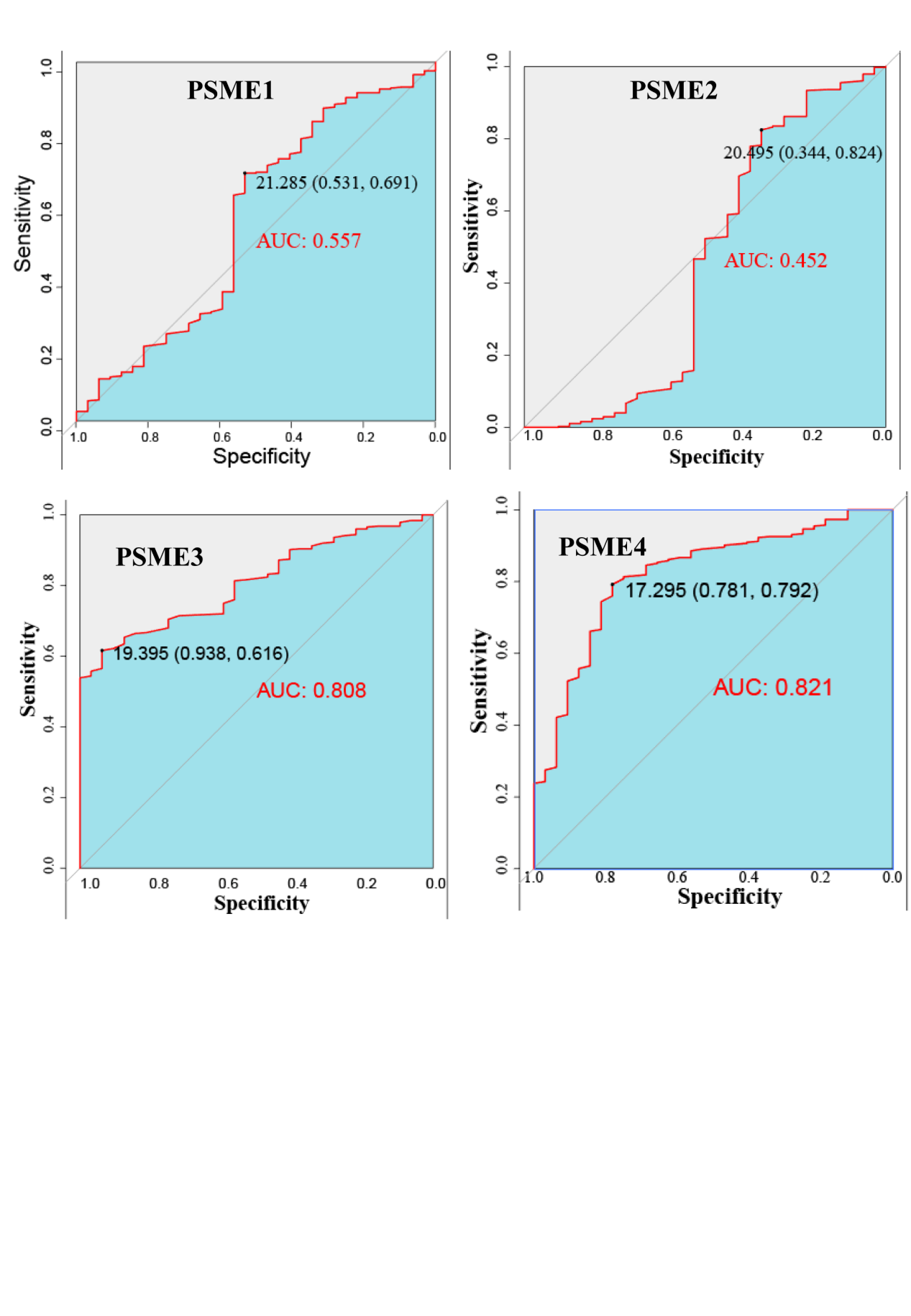
**

**Supplementary Figure 3.** The ROC curve analysis of PSME family genes for distinguishing GC patients from healthy individuals.


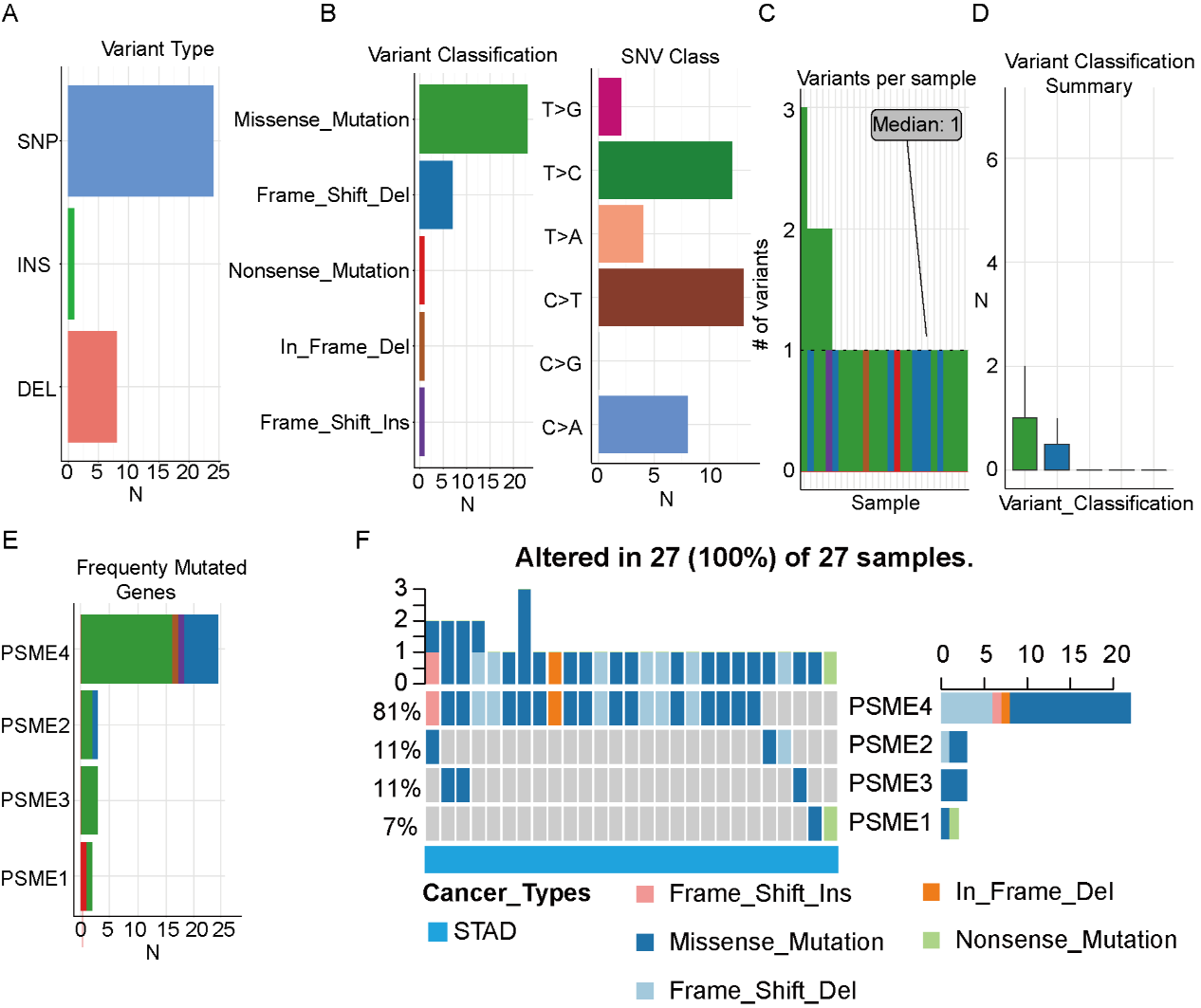


**Supplementary Figure 4.** The single nucleotide variation (SNV) analysis of PSME family genes in STAD (GSCALite).

**A-E**. Summary plot displays SNV frequency and variant types of PSME genes in STAD, and genetic alteration of PSME family genes constitutes SNP, insertion, and deletion. **F**. Waterfall plot shows the mutation distribution of PSME family genes in STAD.


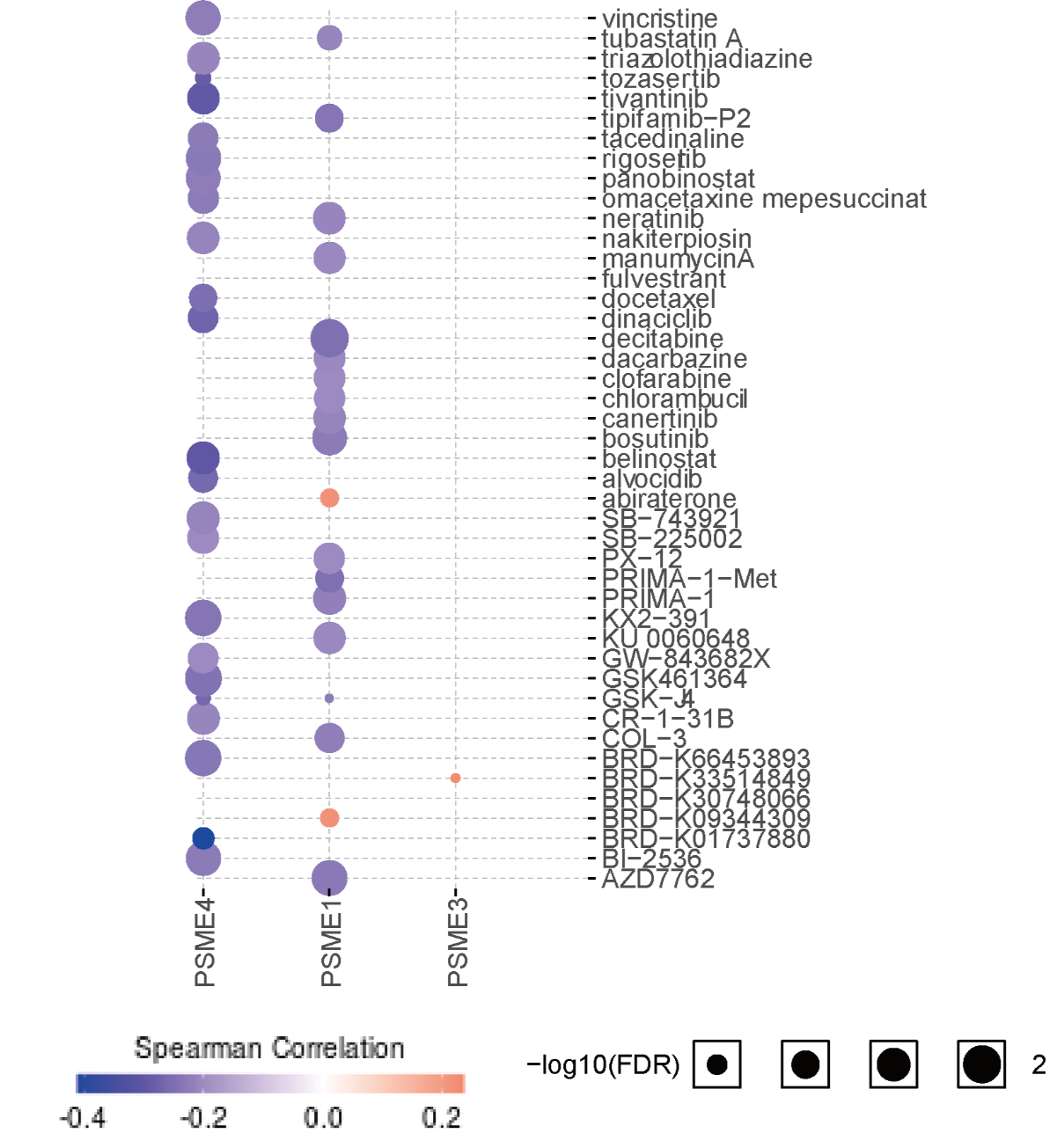


**Supplementary Figure 5.** The drug resistance analysis of PSME family genes based on CTRP drug data (GSCALite). The spearman represents the gene expression correlates with the drug. The positive correlation means that the gene high expression is resistant to the drug, vise verse. Low PSME4 expression level is resistant to 23 drugs or small molecule and low PSME1 level is resistant to 17 drugs or small molecules.


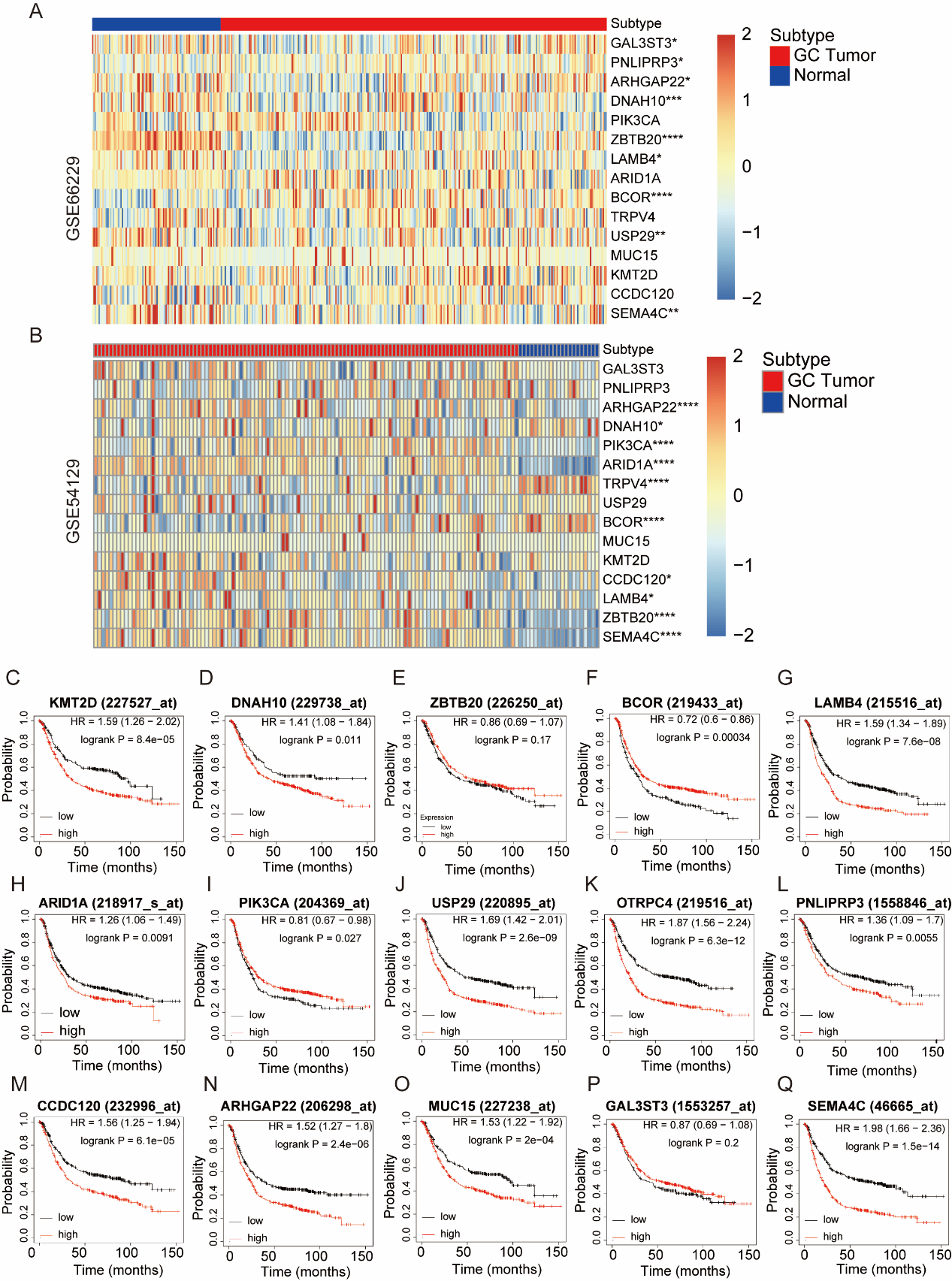


**Supplementary Figure 6.** The expression level and prognostic values of these genes that are significantly pinpoint mutations correlated to PSME family genes’ expression.

A-B: the differences of these genes’ expression between tumor tissues and normal tissues in GSE66229 (A) and GSE54129 (B) dataset, respectively.

C-Q: The relationship between these genes’ expression level and OS in patients with GC.


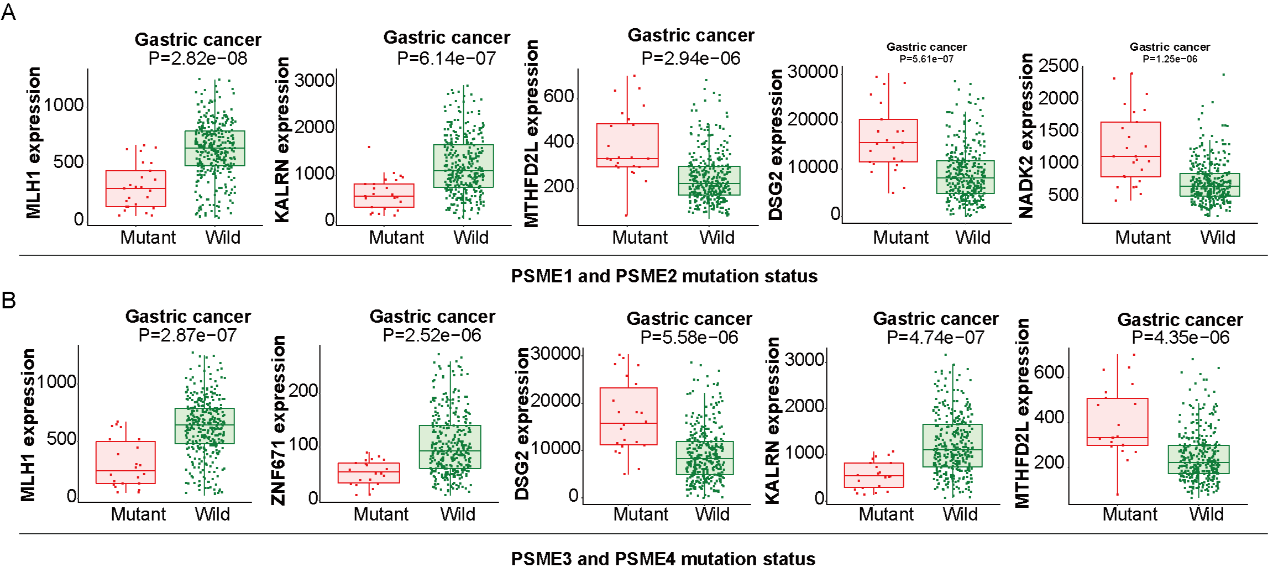


**Supplementary Figure 7.** Linking PSME family genes mutation to gene expression changes in GC.

A-B: The boxplots show top five genes with the strongest dysregulation that are significantly associated with PSME family genes mutation.


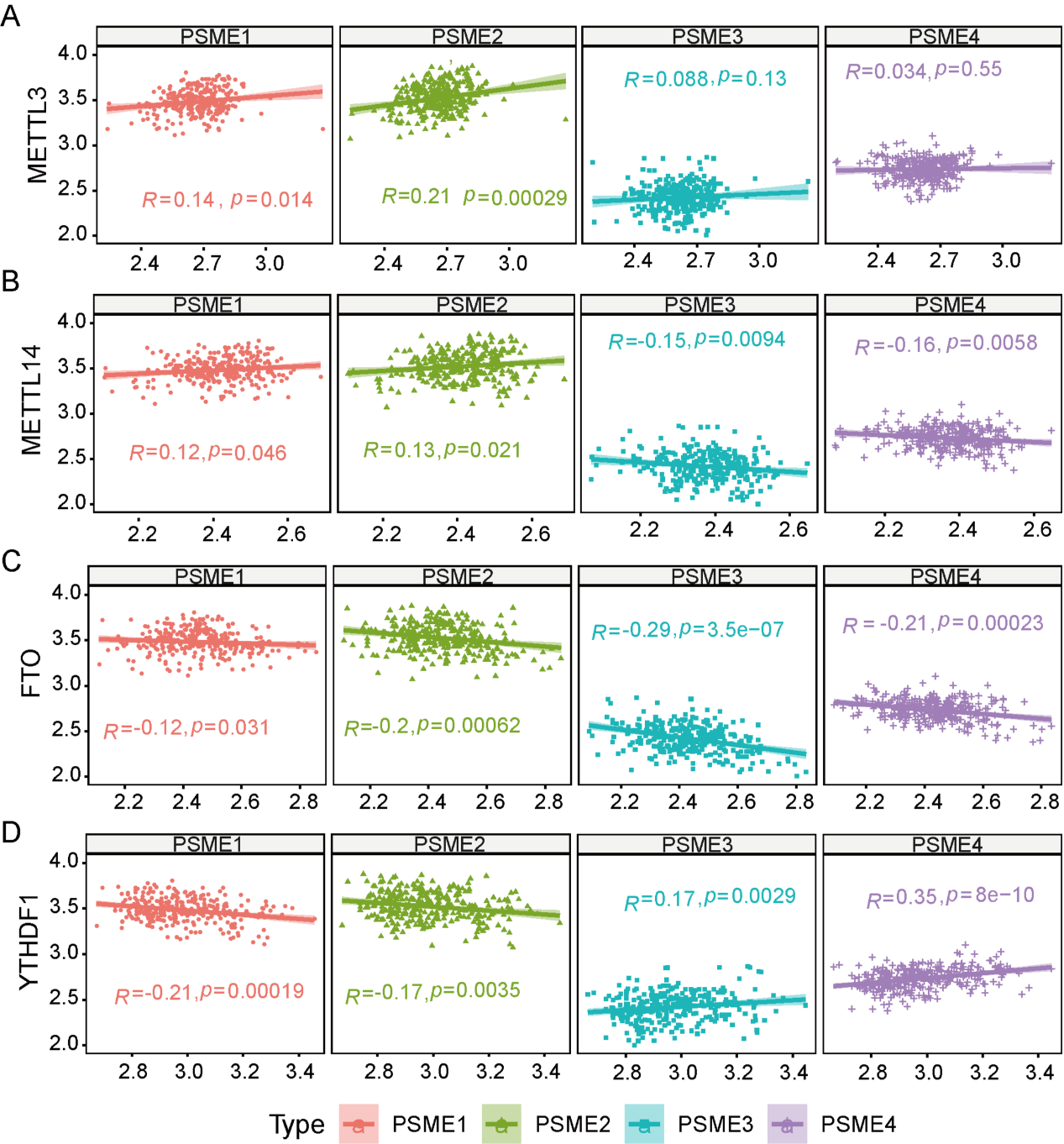


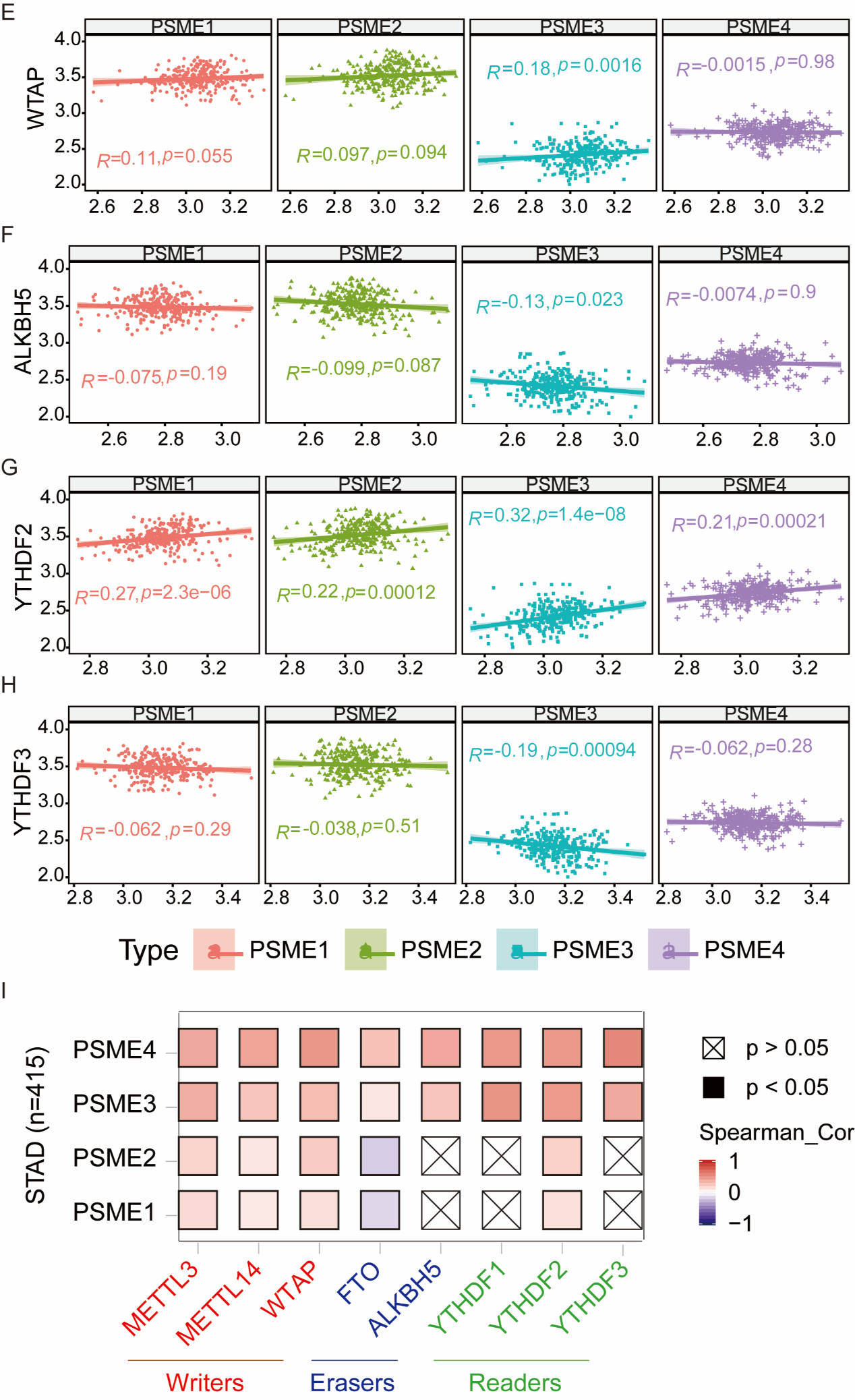


**Supplementary Figure 8.** Correlation between PSME genes expression and m6A related genes in GC.

A-H: Correlation between PSME genes expression and m6A related genes in GSE62254 cohort.

I: Correlation between PSME genes expression and m6A related genes in TCGA-STAD cohort.


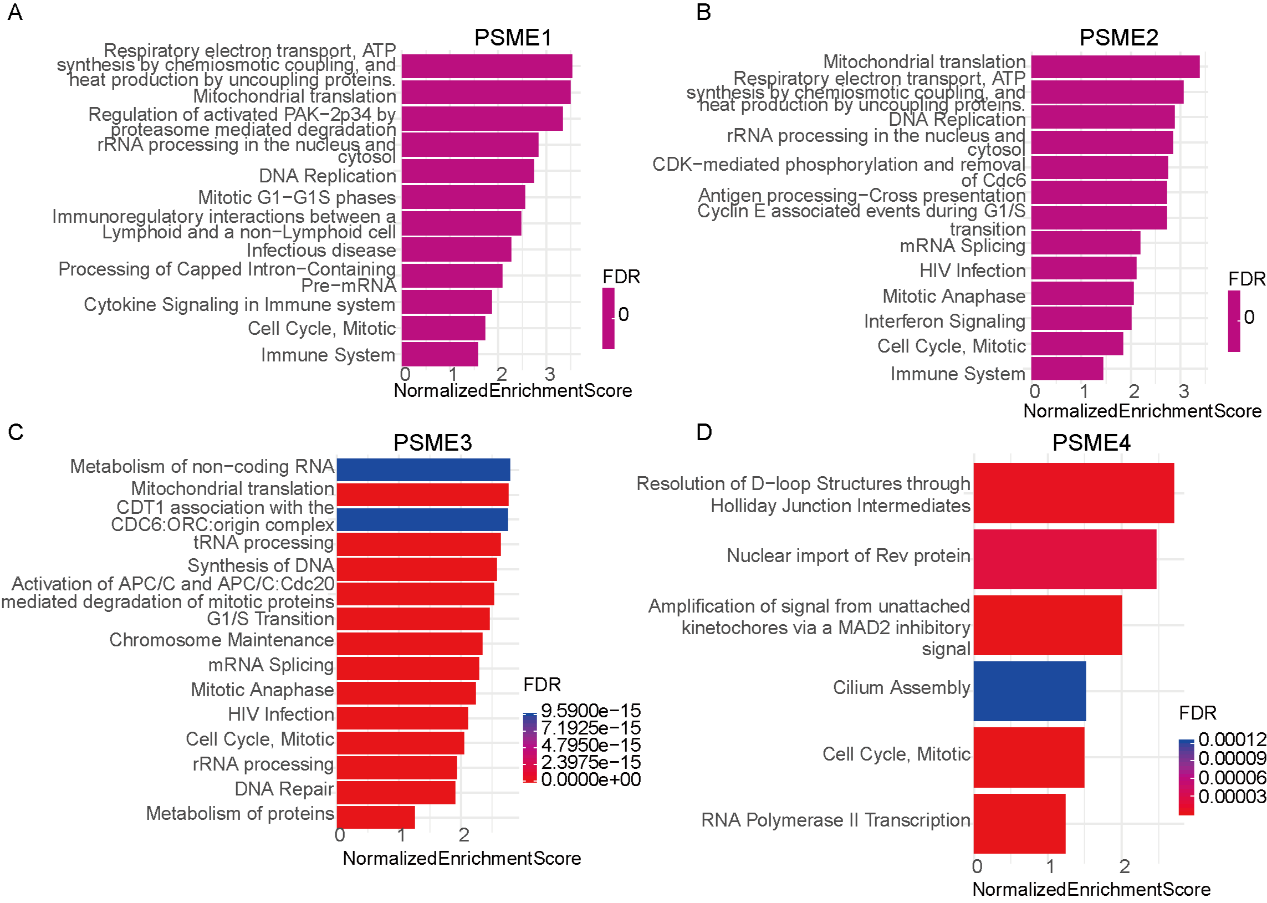


**Supplementary Figure 9**. Reactome pathway analysis of PSME family genes in GC using Linkedomics. Only listed 10 most common functional pathways enriched. FDR was adjusted *P*-values and was set at 0.05. (A): PSME1. (B): PSME2. (C): PSME3. (D): PSME4.

**
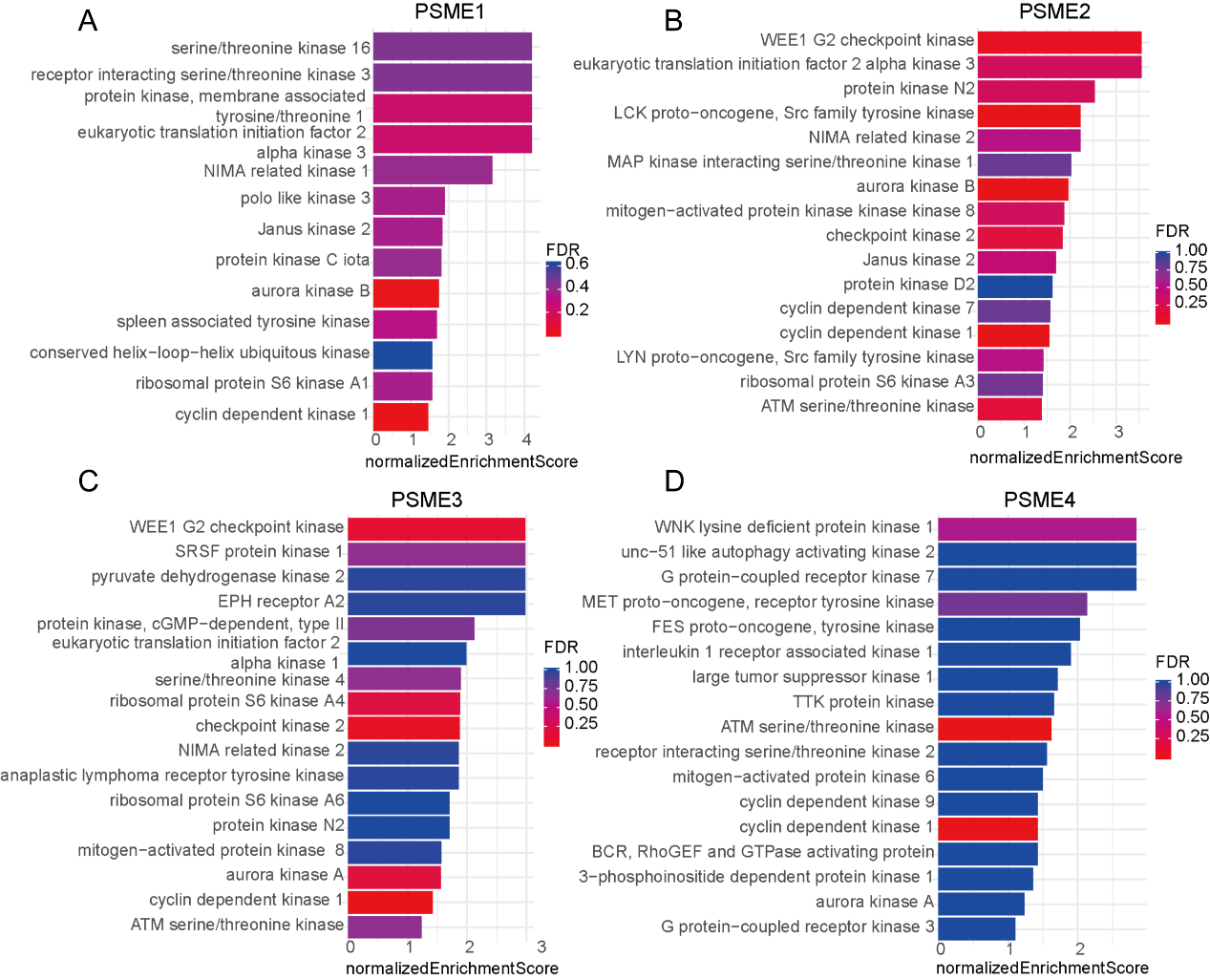
**

**Supplementary Figure 10**. Kinase Target of PSME family genes in GC using linkedomics database. FDR was adjusted *P*-values and was set at 0.05. (A): PSME1. (B): PSME2. (C): PSME3. (D): PSME4.


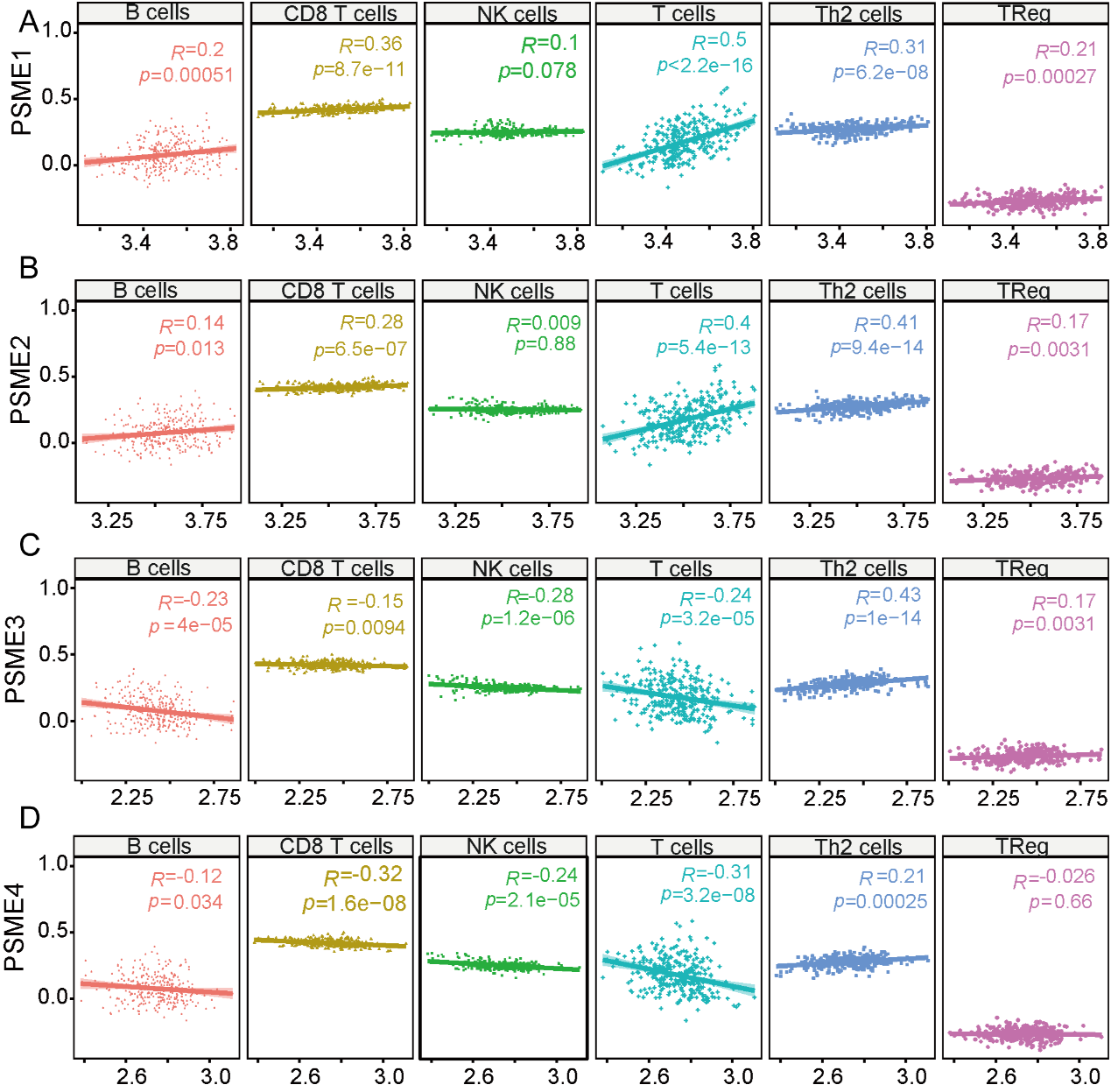


**Supplementary Figure 11.** The correlation of PSME family genes and the infiltration of immune cells. (A): PSME1. (B): PSME2. (C): PSME3. (D): PSME4.


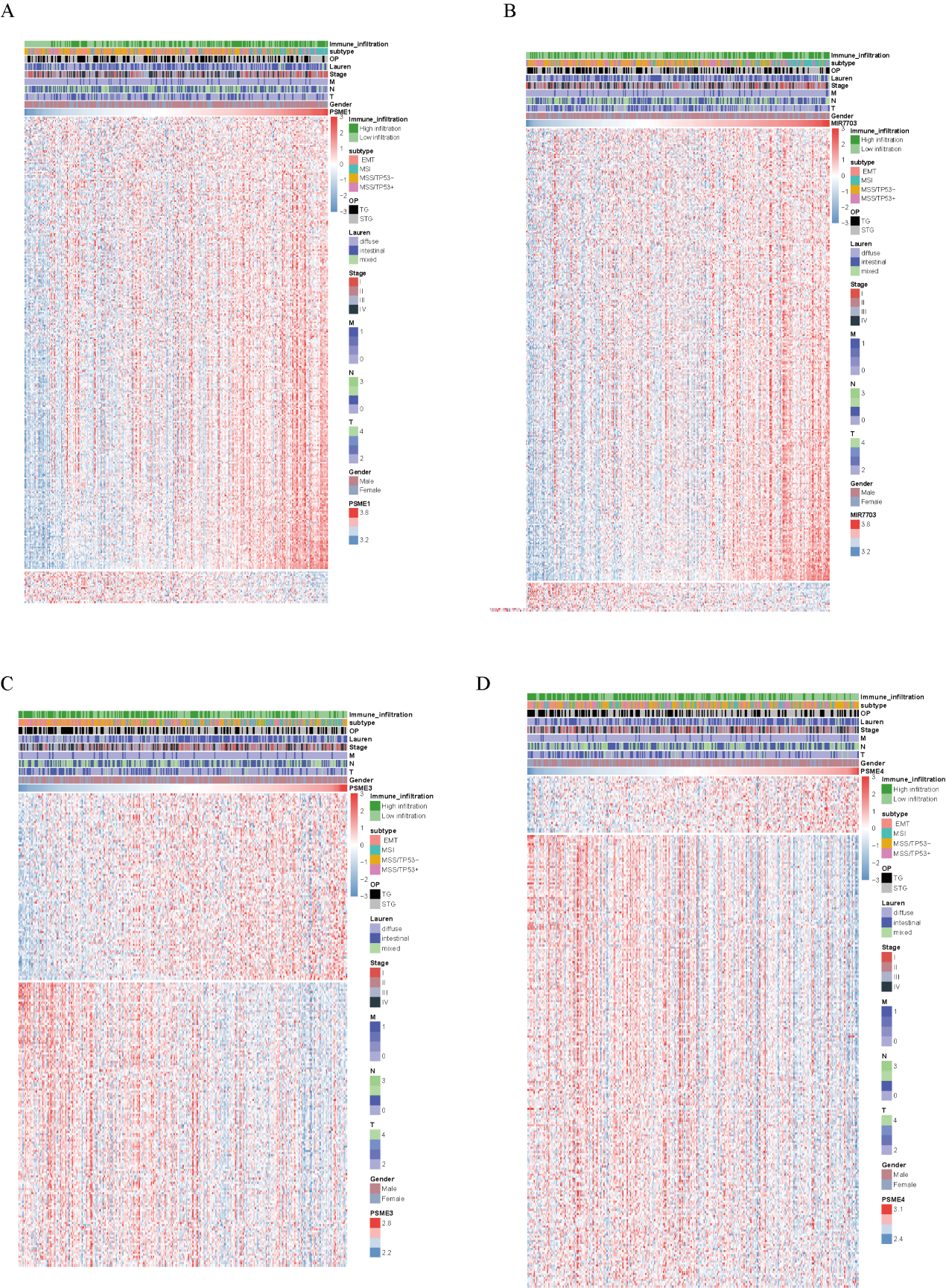


**Supplementary Figure 12.** The correlation between PSME genes expression and 411 immune-related genes in GC. (A): PSME1. (B): PSME2. (C): PSME3. (D): PSME4.


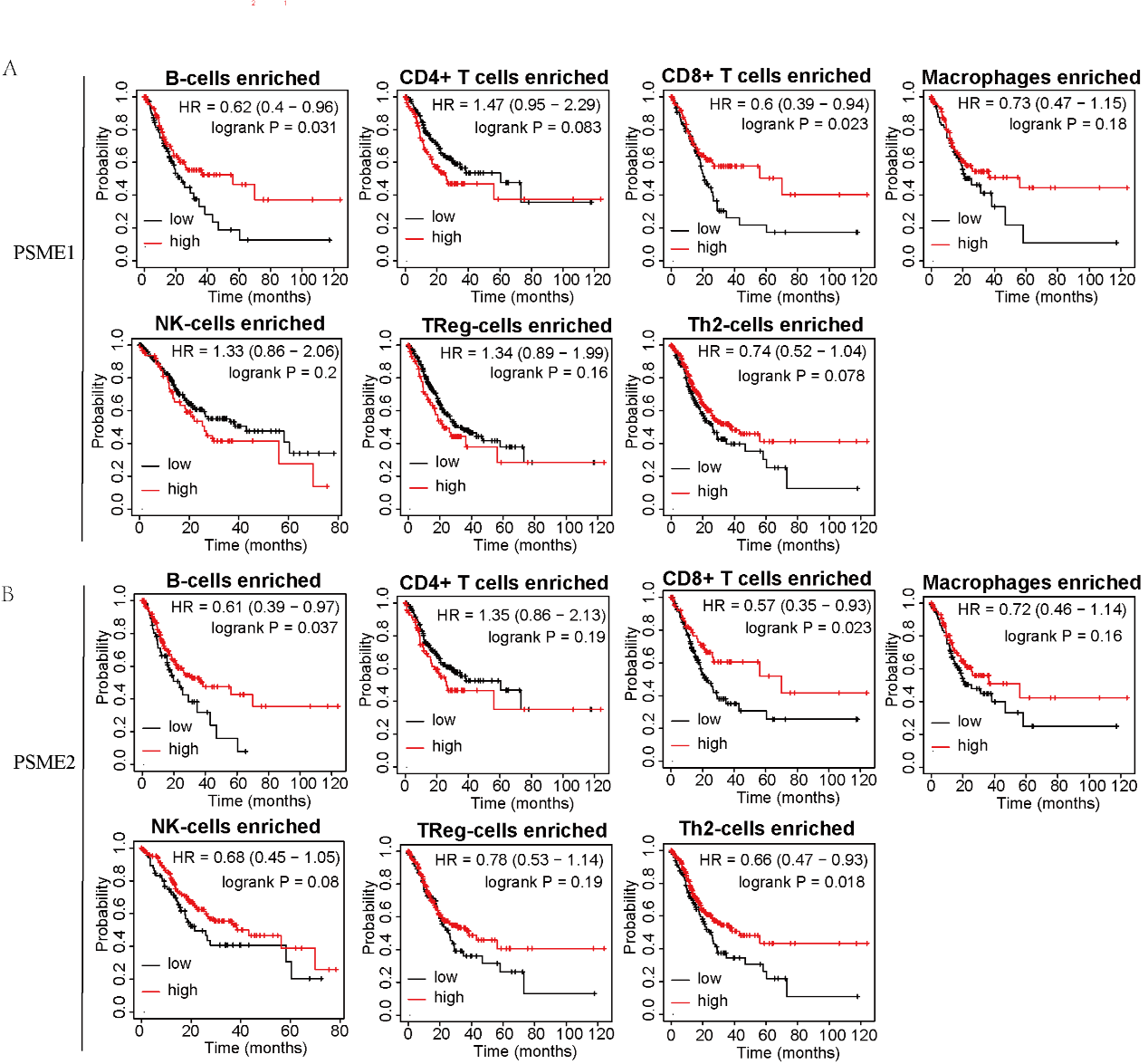


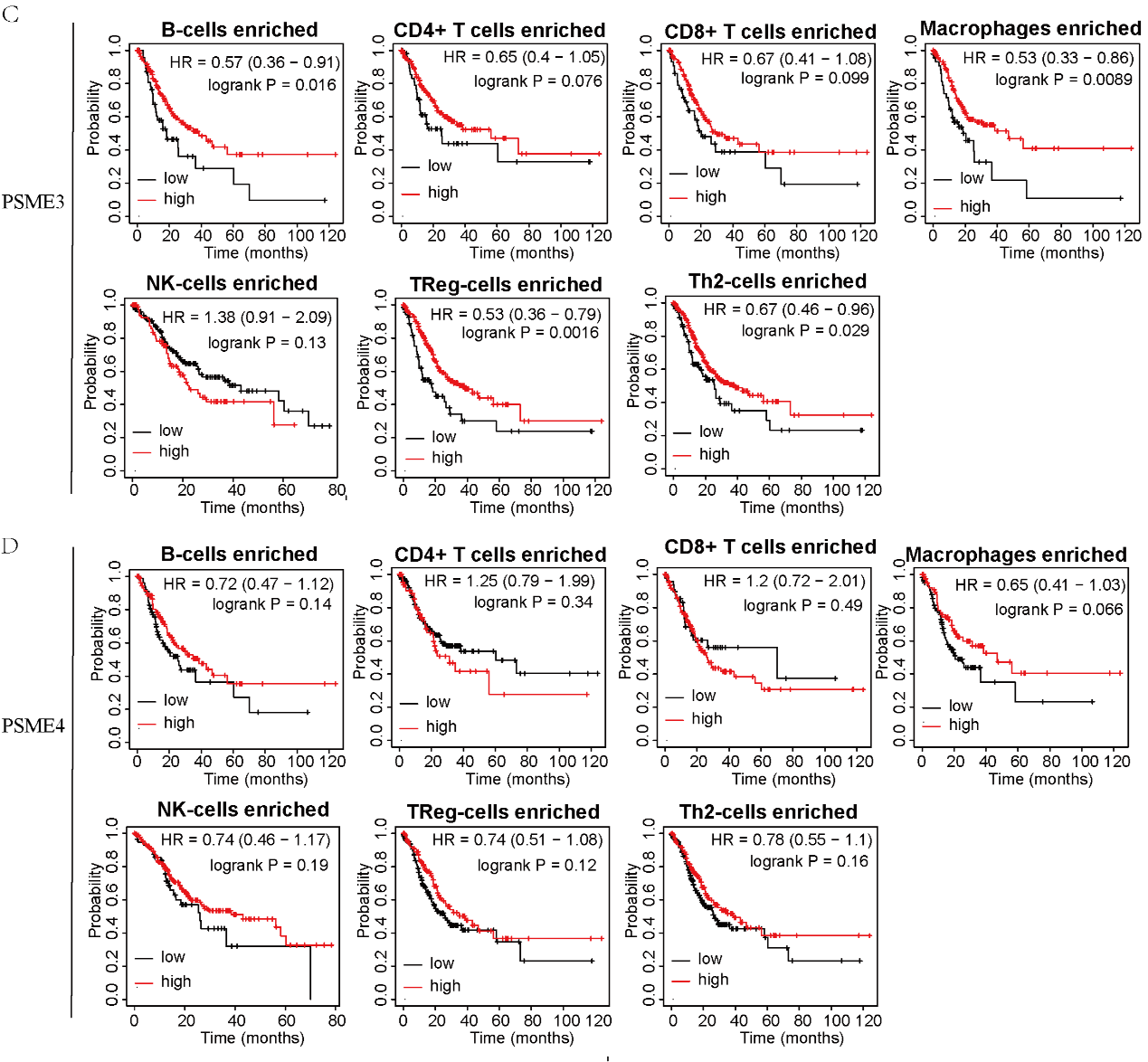


**Supplementary Figure 13.** Relationship between PSME1-4 expression and overall survival in GC patients with different immune cells enriched. (A): PSME1. (B): PSME2. (C): PSME3. (D): PSME4.


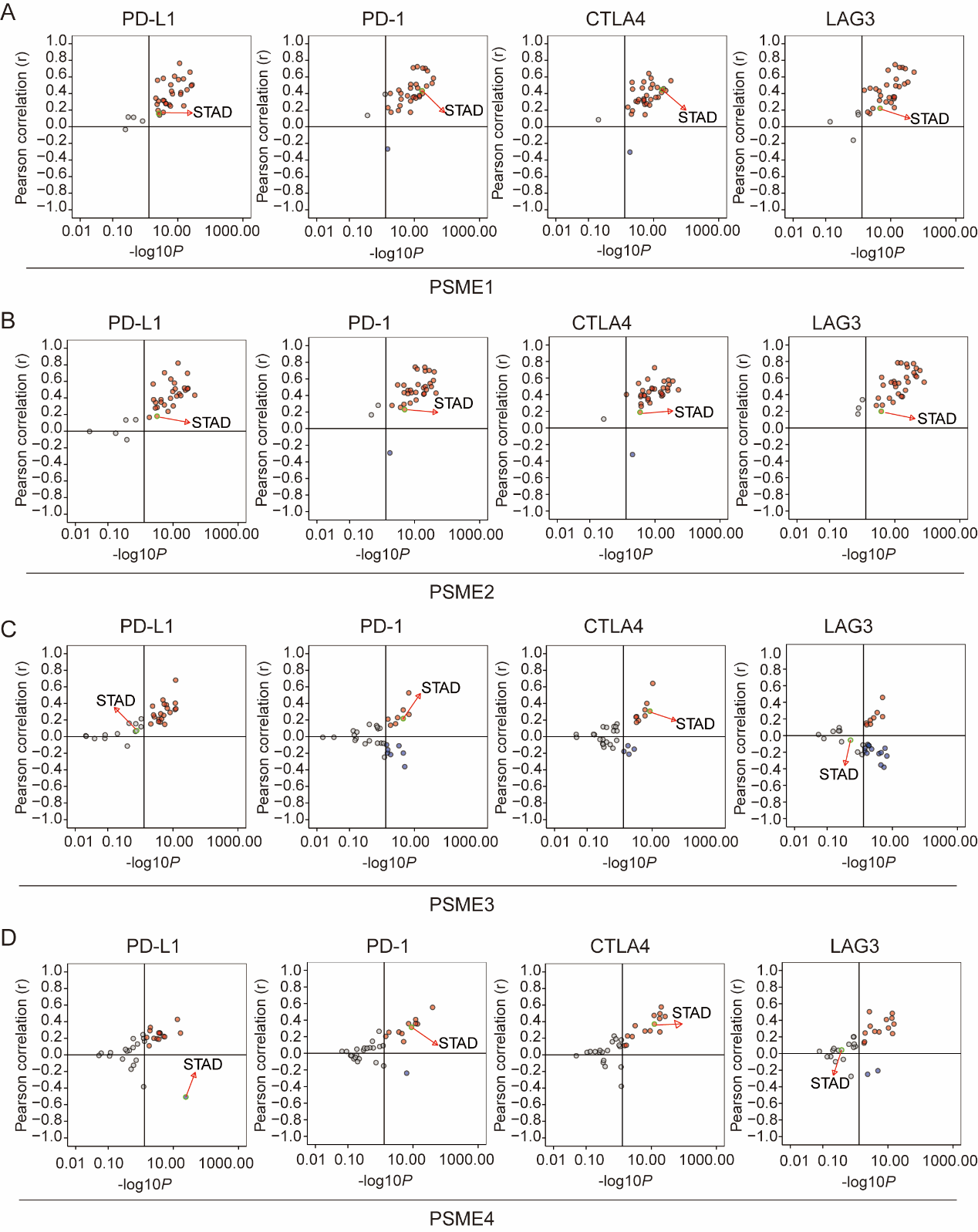


**Supplementary Figure 14.** Correlation between PSME genes expression and PD-L1, PD-1, CTLA4, and LAG3 in 33 cancer types. The dots represent 33 cancer types obtained from TCGA database. Y-axis represents the Pearson correlation and Y-axis represents -log10P.

(A): PSME1. (B): PSME2. (C): PSME3. (D): PSME4.


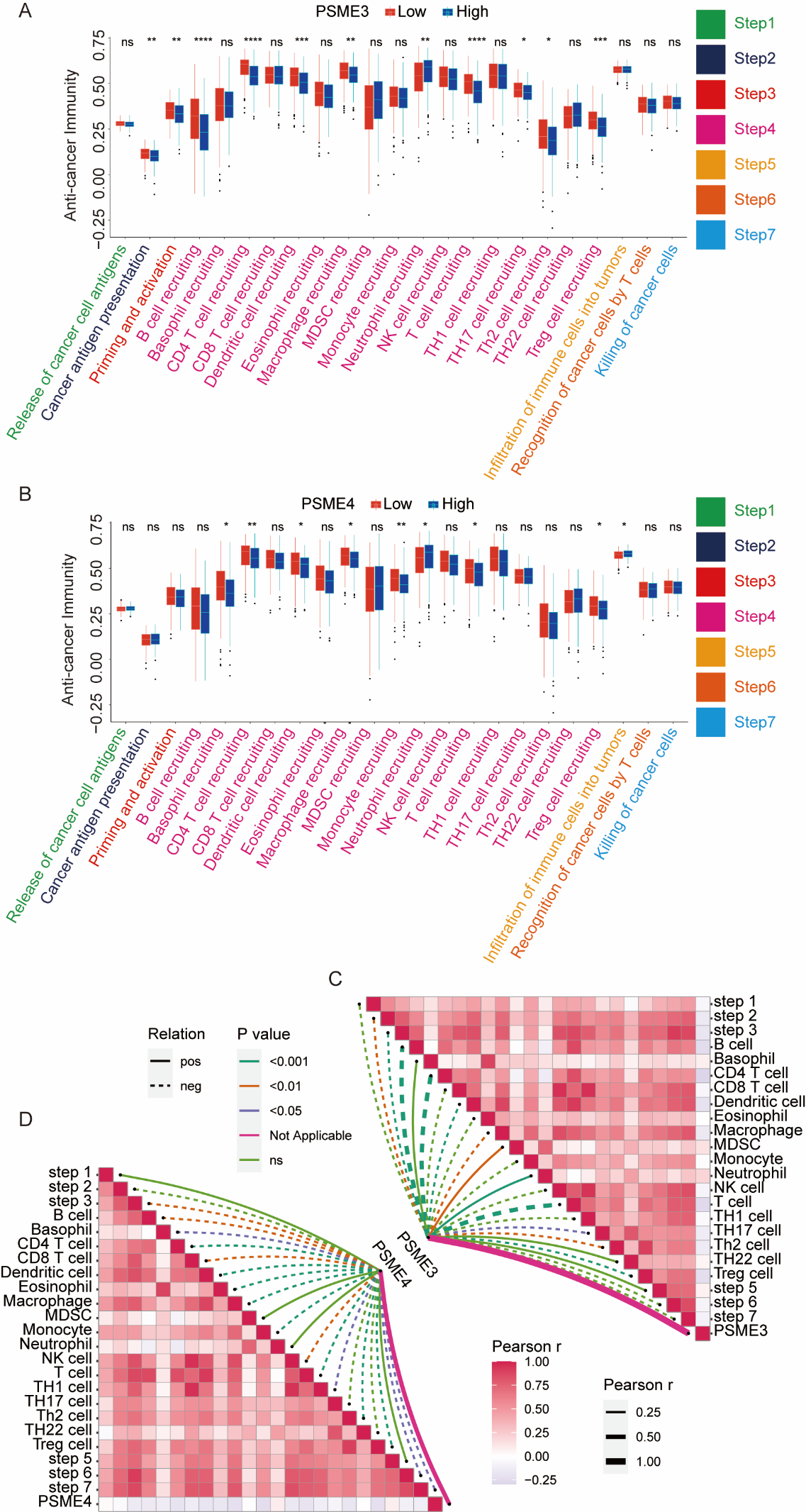


**Supplementary Figure 15.** The relationship between PSME genes and anti-cancer immunity cycle

A: Differences in the multiple steps of the anti-cancer immunity cycle high and low PSME3 groups in GC.

B: Differences in the multiple steps of the anti-cancer immunity cycle high and low PSME4 groups in GC.

C: Correlation between PSME3 and the steps of the anti-cancer immunity cycle.

D: Correlation between PSME4 and the steps of the anti-cancer immunity cycle.

*p<0.05; **p<0.01; ***p<0.001; ****p<0.0001.


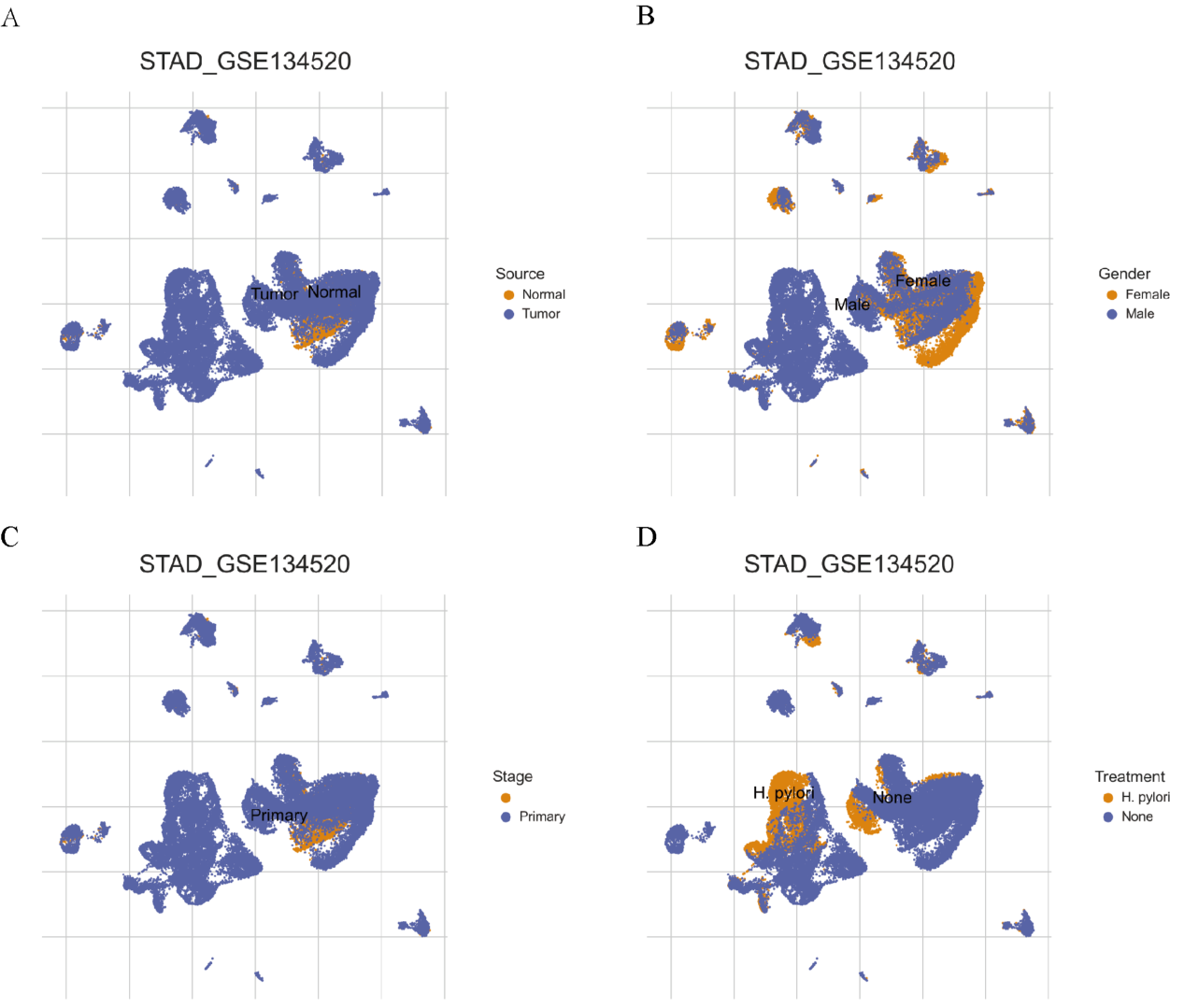


**Supplementary Figure 16.** UMAP visualization of dataset STAD_GSE134520. (A-D) UMAP visualization of dataset STAD_GSE134520, colors represent the major-lineage Source, Gender, Stage, and Treatment, respectively.


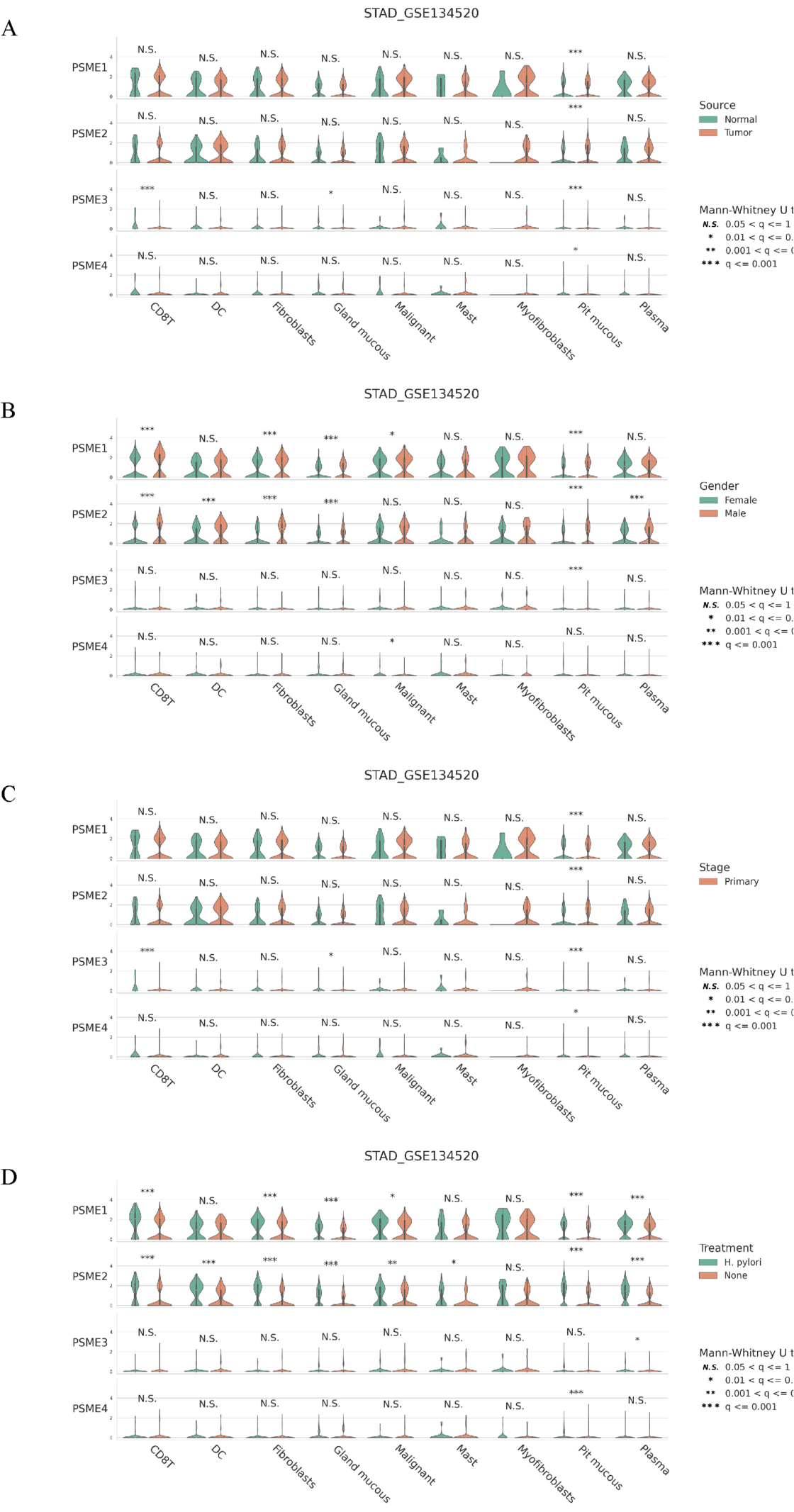


**Supplementary Figure 17.** The distribution of PSME gene expression in different cell types in GC. (A-D) The grid violin plot reflects the distribution of PSME gene expression in different cell types across STAD_GSE134520 dataset with different source, gender, stage, and treatment, respectively.
