# Supplementary material for: The Expression Patterns and Prognostic Value of the Proteasome Activator Subunit Gene Family in Gastric Cancer Based on Integrated Analysis
Source: Front Cell Dev Biol. 2021 Sep 28;9:663001. doi: 10.3389/fcell.2021.663001 (PMC8505534; doi:10.3389/fcell.2021.663001)
Supplement: Supplementary file 2 [file Data_Sheet_2.docx]

**The expression patterns and prognostic value of the proteasome activator subunit gene family in gastric cancer based on integrated analysis**

**Yongdong Guo^1^, Xiaoping Dong^1^, Jing Jin^1^, Yutong He^1^**

**1 Cancer Institute, Fourth Hospital of Hebei Medical University, Shijiazhuang, 050011, China**

**Correspondence should be addressed to: Yutong He, (E-mail: 15733291685@163.com)**

**Short Title: Proteasome activator subunit genes as predictive gastric cancer markers**

**Table S1**. Clinical features of patients with GC (n = 40).

| Clinicopathological parameters | Group | Number of cases (%) |
| --- | --- | --- |
| Sex | Male | 29 (72.50%) |
|  | Female | 11 (27.50%) |
| Age (years) | ≥65 | 18 (45.00%) |
|  | <65 | 22 (55.00%) |
| Tumor size (cm) | <5 | 24 (60.00%) |
|  | ≥5 | 16 (40.00%) |
| Histologic differentiation | Well or moderate | 12 (30.00%) |
|  | Poor | 28 (70.00%) |
| TNM stage | Ⅰ+Ⅱ | 19 (47.50%) |
|  | Ⅲ‐Ⅳ | 21 (52.50%) |
| Distant metastasis | Absent | 17 (42.50%) |
|  | Present | 23 (57.50%) |

**Table S2.** The primers of PSME family genes and GAPDH

| Gene |  | Sequence |
| --- | --- | --- |
| GAPDH | F | 5′-CCACCCATGGCAAATTCC-3′ |
|  | R | 5′-GATGGGATTTCCATTGATGACA-3′ |
| PSME1 | F | 5′ –GCGCTTGAAGCCTGAGATCA-3′ |
|  | R | 5′-CCTTCTCCTGGACAGCCACT-3′ |
| PSME2 | F | 5'-CTTTTCCAGGAGGCTGAGGAAT-3' |
|  | R | 5‘-GGAGGGAAGTCAAGTCAGCC-3’ |
| PSME3 | F | 5′-CCAGACCTAAGCTGCCTTCT-3′ |
|  | R | 5′-GATAGCAGCCTCTACTGGCA-3′ |
| PSME4 | F | 5'-GGACATGCTTGGTGTAGCCT- 3' |
|  | R | 5'- GGGCATCTCGGTAGTACAGC-3' |

Abbreviations: PSME, proteasome activator subunit; GAPDH, glyceraldehyde-3-phosphate dehydrogenase.

**Table S3.** Details of GEO datasets

| **Series** | **Platform** | **Country** | **Year** | **Number of Tumor tissues（n）** | **Number of normal tissues（n）** |
| --- | --- | --- | --- | --- | --- |
| **GSE29272** | GPL96 | USA | 2011 | 134 | 134 |
| **GSE66229** | GPL570 | USA | 2015 | 300 | 100 |
| **GSE62254** | GPL570 | USA | 2014 | 300 | 0 |
| **GSE54129** | GPL570 | China | 2014 | 111 | 21 |

**Table S4.** The number of various RNA modifications of PSME family genes based on RMBase v2.0 database.

| Gene | Gene ID | Strand | m6A Num | m1A Num | m5C Num | 2’-O-Me Num | PseudoU Num | Other Num | Total Num |
| --- | --- | --- | --- | --- | --- | --- | --- | --- | --- |
| PSME1 | ENSG00000131467.10 | + | 11 | 0 | 0 | 0 | 1 | 0 | 12 |
| PSME2 | ENSG00000100911.13 | - | 12 | 0 | 0 | 0 | 0 | 0 | 12 |
| PSME3 | ENSG00000092010.14 | + | 12 | 0 | 0 | 1 | 0 | 0 | 13 |
| PSME4 | ENSG00000068878.14 | - | 11 | 0 | 0 | 1 | 0 | 0 | 12 |

**Table S5**. Differentially expressed PSME genes in each cluster. PSME1 and PSME2 may be cell-type’s potential markers.

| Cluster | Celltype (malignancy) | Celltype (major-lineage) | Celltype (minor-lineage) | Gene | Log2 FC | Percentage (%) | Adjusted  *P*-value |
| --- | --- | --- | --- | --- | --- | --- | --- |
| 1 | Stromal cells | Pit mucous | Pit mucous | PSME1 | -0.3 | 25.6 | 2.7E-132 |
| 2 | Stromal cells | Gland mucous | Gland mucous | PSME1 | -0.31 | 44.2 | 3.33E-37 |
| 13 | Immune cells | CD8T | CD8Tcm | PSME1 | 0.49 | 46.5 | 1.22E-15 |
| 14 | Stromal cells | Pit mucous | Pit mucous | PSME1 | 0.43 | 82.4 | 1.52E-136 |
| 16 | Stromal cells | Fibroblasts | Fibroblasts | PSME1 | 0.28 | 51.8 | 7.59E-12 |
| 18 | Malignant cells | Malignant | Malignant | PSME1 | 0.36 | 56.9 | 8.31E-20 |
| 22 | Stromal cells | Myofibroblasts | Myofibroblasts | PSME1 | 0.53 | 53 | 3.84E-06 |
| 0 | Stromal cells | Pit mucous | Pit mucous | PSME2 | -0.37 | 29.1 | 1.00E-142 |
| 1 | Stromal cells | Pit mucous | Pit mucous | PSME2 | -0.62 | 19 | 3.42E-242 |
| 2 | Stromal cells | Gland mucous | Gland mucous | PSME2 | -0.53 | 37.5 | 6.63E-82 |
| 3 | Stromal cells | Pit mucous | Pit mucous | PSME2 | 0.78 | 74.1 | 2.08E-287 |
| 7 | Stromal cells | Gland mucous | Gland mucous | PSME2 | -0.44 | 43.5 | 2.78E-26 |
| 8 | Stromal cells | Pit mucous | Pit mucous | PSME2 | -0.62 | 33.9 | 3.23E-72 |
| 9 | Stromal cells | Pit mucous | Pit mucous | PSME2 | 0.27 | 74.9 | 5.17E-106 |
| 10 | Stromal cells | Pit mucous | Pit mucous | PSME2 | 0.26 | 59.7 | 1.28E-38 |
| 12 | Stromal cells | Pit mucous | Pit mucous | PSME2 | 0.41 | 75.3 | 1.42E-113 |
| 14 | Stromal cells | Pit mucous | Pit mucous | PSME2 | 1.09 | 92.1 | 0 |
| 20 | Immune cells | Mast | Mast | PSME2 | -0.45 | 24 | 6.30E-16 |
